# Defect and Interface Engineering of VO_2_ for Reconfigurable Nanophotonics

**DOI:** 10.3390/nano16181132

**Published:** 2026-09-10

**Authors:** Ardak Ainabayev, Zinetula Insepov, Kurbangali Tynyshtykbayev

**Affiliations:** 1 Institute of Physics and Technology, Satbayev University, Almaty 050032, Kazakhstan; kurbangali.tnyshtykbayev@nu.edu.kz; 2 Nazarbayev University Research Administration, Nazarbayev University, Astana 010000, Kazakhstan; 3 School of Physics and Centre for Research on Adaptive Nanostructures and Nanodevices (CRANN), Trinity College Dublin, D02 PN40 Dublin, Ireland; 4 School of Nuclear Engineering, Purdue University, West Lafayette, IN 47907, USA

**Keywords:** correlated oxides, oxygen nonstoichiometry, heterointerfaces, active optics, thermochromic switching, adaptive infrared devices, heterogeneous integration

## Abstract

Vanadium dioxide (VO_2_) is a prominent active material for reconfigurable nanophotonics because its reversible metal-insulator transition produces large changes in complex refractive index and electrical conductivity. The usable phase contrast, however, is not an intrinsic constant: it is governed by defect type and location, vanadium valence, oxygen stoichiometry, strain, crystallographic orientation, dimensionality, and the chemical, electrical, optical, and thermal boundary conditions imposed by interfaces. This focused narrative review develops a defect- and interface-centred framework linking VO_2_ phase physics to device-level optical modulation. Bulk, surface, grain-boundary, and heterointerface defects are distinguished, together with their effects on carriers, V-V bonding, phase stability, optical loss, and cycling reliability. Epitaxial and polycrystalline films, ultrathin layers, and nanostructures are compared across the visible, near-infrared, mid-infrared, and terahertz ranges. Thermal, optical, electrical, electrostatic, electrochemical, ionic, strain, and ferroelectric activation pathways are then compared according to volatility, speed, retention, reversibility, and endurance. Representative free-space metasurfaces, guided-wave modulators, adaptive emitters, and photonic memories are benchmarked separately to avoid mixing incomparable performance definitions. The resulting analysis shows that optical modulation, insertion loss, thermal overhead, ambient stability, and endurance are coupled through the same defect and interface landscape. Progress, therefore, requires coordinated control of phase purity, local chemistry, interface energetics, thermal transport, and architecture-specific performance reporting.

## 1. Introduction

Nanophotonic structures provide precise control over the amplitude, phase, polarisation, spectrum, and propagation direction of light at deeply subwavelength length scales. Metasurfaces, nanoantennas, photonic resonators, and integrated waveguides have consequently enabled compact optical components with functionalities that are difficult to obtain using conventional bulk optics. However, the electromagnetic response of most nanophotonic structures is fixed by their geometry and material composition during fabrication. This restricts their ability to adapt to changing operating conditions or perform multiple optical functions within a single device. The development of active materials whose optical properties can be reversibly modified by external stimuli is therefore central to reconfigurable nanophotonics [1].

Phase-transition materials are particularly attractive for this purpose because a relatively small stimulus can induce substantial changes in their complex refractive index and electrical conductivity [2]. Among these materials, vanadium dioxide (VO_2_) occupies a distinctive position. Unlike chalcogenide phase-change materials, which commonly rely on switching between amorphous and crystalline states, VO_2_ undergoes a reversible and generally volatile transition between insulating and metallic phases without requiring melting and recrystallisation [2,3]. The transition can be activated thermally, electrically, optically, mechanically, or through ionic and electrochemical processes, allowing VO_2_ to be incorporated into devices with different actuation and integration requirements [3].

At atmospheric pressure, stoichiometric VO_2_ undergoes a first-order metal-insulator transition at approximately 68 °C [4]. This transition is generally accompanied by a structural transformation from a low-temperature monoclinic M1 phase to a high-temperature tetragonal rutile phase. In the monoclinic phase, the pairing and tilting of vanadium atoms contribute to the formation of an insulating electronic state, whereas the rutile phase exhibits metallic conductivity [5]. The relative contributions of electron correlation and lattice distortion to this transition remain actively discussed, and spatially resolved infrared measurements have revealed nanoscale metallic-domain nucleation and phase coexistence across the transition [6]. From a photonic perspective, the most important consequence is the pronounced modification of the complex dielectric function, particularly in the near-infrared, mid-infrared, and terahertz spectral regions [7]. This optical contrast allows the resonance condition and electromagnetic-field distribution of a VO_2_-containing nanostructure to be altered dynamically.

These properties have stimulated the development of VO_2_-based tunable absorbers, optical switches, modulators, polarisation-control elements, thermal emitters, photodetectors, and reconfigurable metasurfaces [3]. For example, nanoscale defect patterning has been used to create active optical metasurfaces with thermally controlled absorption and polarisation responses [8]. Electrically tunable VO_2_ metasurfaces have demonstrated substantial optical phase modulation [9], while epitaxial VO_2_ nanostructures have been explored as large-area switchable dielectric resonators [10]. More recent designs combine VO_2_ with metal-insulator-metal resonators, local heaters, and programmable control schemes to enable functions such as dynamic beam steering [11]. These studies show that VO_2_ can provide more than binary transmission switching: through the coexistence of insulating and metallic domains, it can also support intermediate optical states and continuously tunable responses.

Nevertheless, the performance of VO_2_ nanophotonic devices is not determined solely by the nominal dielectric functions of ideal monoclinic and rutile VO_2_. The transition temperature, hysteresis width, optical contrast, switching threshold, phase homogeneity, and cycling stability depend strongly on the material’s defect chemistry and microstructure. Oxygen vacancies can introduce electron carriers, modify local V-V bonding, promote metallic behaviour, and partially or completely suppress the insulating phase [12,13]. Conversely, excess oxygen can favour the formation of V_2_O_5_ or other vanadium oxide phases, particularly at surfaces and grain boundaries. Grain size, crystallographic defects, residual stress, and compositional non-uniformity can further broaden the transition by producing spatially distributed nucleation temperatures. Although these effects are sometimes treated simply as fabrication imperfections, controlled defect engineering offers a route for locally programming the optical response, lowering the switching temperature, narrowing or broadening the hysteresis, and stabilising intermediate states.

Interfaces introduce an additional level of control. The substrate determines the crystallographic orientation, epitaxial strain, grain structure, and, in some cases, the optical anisotropy of the VO_2_ layer [14]. At the device level, thermal boundary resistance and the thermal conductivity of the substrate and surrounding dielectric layers govern the switching energy, response time, heat dissipation, and thermal crosstalk between neighbouring elements. Film thickness is similarly important because ultrathin VO_2_ can provide reduced optical loss and lower thermal mass, but interface disorder, strain relaxation, and discontinuous growth can weaken or broaden the transition. Epitaxial growth on c-plane Al_2_O_3_ has demonstrated that thin VO_2_ layers can retain a pronounced transition with suppressed hysteresis when the growth conditions and interfacial structure are carefully controlled [15]. Scalable deposition methods, including spray pyrolysis, can also produce epitaxial VO_2_ with tunable transition characteristics, indicating that high-quality phase-transition films need not be restricted to vacuum-based fabrication [16].

A central challenge is therefore to establish direct causal relationships between VO_2_ synthesis, the type and location of defects, interfacial boundary conditions, phase-transition behaviour, optical constants, and final device performance. Earlier reviews provide essential but complementary perspectives. Shao et al. emphasised phase-transition mechanisms and routes for modulating the transition [17]; Liu et al. surveyed the physics, dynamics, phase diagrams, imperfection effects, and broad applications of VO_2_ [18]; Cueff et al. focused specifically on the emerging field of VO_2_ nanophotonics [3]; Zhang et al. organised the literature around VO_2_ polymorphs, fabrication of nanostructures, properties, and applications [19]; Bhupathi et al. reviewed multi-stimulus control and the associated device landscape [20]; and Wang et al. recently centred the discussion on switching dynamics in optoelectronic devices [21]. The present review complements these works by using the spatial location of defects and the function of interfaces as the organising variables that connect material processing to optical loss, threshold, speed, retention, and endurance. It also separates free-space metasurfaces, guided-wave devices, thermal emitters, and photonic memories so that dissimilar performance definitions are not combined. Table 1 summarises these differences in scope and emphasis.

### Scope and Literature-Selection Approach

This article is a focused narrative review rather than a systematic meta-analysis. Foundational studies from 1959 onward were retained to establish the structural and electronic physics of VO_2_, while the device survey emphasised peer-reviewed experimental literature available through August 2026. Literature was identified using Google Scholar and publisher databases, supplemented by backward and forward citation searches from seminal papers and recent reviews. Search terms combined “VO_2_” or “vanadium dioxide” with “metal-insulator transition”, “oxygen vacancy”, “defect”, “strain”, “interface”, “doping”, “metasurface”, “modulator”, “thermal emitter”, “photonic memory”, and related variants. Priority was given to studies that reported temperature-dependent structural, chemical, electrical, or optical characterisation and to device papers that quantified at least one architecture-relevant metric. Purely numerical designs without experimental validation were used only for physical context, while studies lacking a clear material state, activation condition, or performance definition were not used for quantitative benchmarking. The resulting scope covers bulk and single-crystal VO_2_ where they clarify phase mechanisms, but emphasises epitaxial and polycrystalline films, ultrathin layers, and nanostructures relevant to reconfigurable nanophotonics.

## 2. Optical Properties of VO_2_

### 2.1. Metal-Insulator Transition

The optical response of VO_2_ is governed by its first-order metal-insulator transition (MIT). Here, MIT denotes the reversible transition between insulating and metallic states; when the direction matters, “insulator-to-metal on heating” or “metal-to-insulator on cooling” is stated explicitly. In stoichiometric bulk VO_2_, heating through approximately 340 K (68 °C) transforms the low-temperature monoclinic M1 phase into the high-temperature tetragonal rutile R phase [4,5]. In M1, all V ions are paired and tilted along the rutile $c_{R}$ direction. In the monoclinic M2 phase, which can be stabilised by uniaxial stress or dopants such as Cr, only one set of V chains is dimerised, while the other contains tilted but unpaired V ions. A triclinic T phase can interpolate between M1 and M2, and mixed or intermediate states can occur in strained, doped, defective, or spatially nonuniform samples [22,23]. These phases are physically distinct and should not be described as a single broadened M1-R transition.

The equilibrium M1-R transition combines a rearrangement of the V-V chains with a major redistribution of electronic states: the approximately 0.6 eV gap of the insulating phase closes and a strong free-carrier response develops [7]. The designation “metallic” is supported by several mutually consistent observables rather than by crystal symmetry alone: the resistivity decreases by several orders of magnitude, the low-frequency conductivity acquires a finite Drude-like response, the optical gap closes, and the infrared dielectric response becomes dominated by free carriers [5,7]. Rutile symmetry is therefore strongly associated with the equilibrium metallic state, but a structural label should not be used as the sole proof of metallic transport under nonequilibrium excitation.

The MIT is not spatially homogeneous in a real thin film. Metallic domains nucleate within the insulating matrix and grow until a connected metallic network is formed [6,24]. The measured optical response within the transition region is therefore an effective response of coexisting phases rather than that of a uniform intermediate crystal structure. In addition, the transition occurs at different temperatures during heating and cooling, producing hysteresis in the transmittance, reflectance, emissivity, refractive index, and extinction coefficient [25]. The transition temperature and hysteresis width reported for a nanophotonic device must therefore be associated with a specified wavelength, polarisation, thermal branch, and sample geometry.

Under ultrafast optical excitation, electronic and structural observables need not evolve simultaneously. Carrier redistribution and optical-gap collapse can precede the loss of V-V dimer and zigzag order and the eventual development of tetragonal symmetry. Mega-electron-volt ultrafast electron diffraction on quasi-single-crystalline films found that dimer/zigzag order disappeared within approximately 200 fs, whereas tetragonal symmetry developed over approximately 5 ps [26]. These observations support strong electron-lattice coupling without requiring strict simultaneity far from equilibrium. A fast reflectivity or conductivity change should consequently be reported as an electronic response unless simultaneous diffraction or another phase-specific structural probe confirms conversion to the rutile lattice.

### 2.2. Temperature-Dependent Refractive Index and Extinction Coefficient

The optical constants of VO_2_ are commonly expressed through the complex refractive index

(1) $$\tilde{n}\left(\lambda ,T\right)=n\left(\lambda ,T\right)+i\kappa \left(\lambda ,T\right),$$

where *n* is the refractive index, and κ is the extinction coefficient. The corresponding complex relative permittivity is

(2) $$\tilde{\varepsilon }\left(\lambda ,T\right)={\tilde{n}}^{2}=\left(n^{2}-\kappa^{2}\right)+i\;2n\kappa ,$$

and the absorption coefficient is α=4πκ/λ. These relations show that both the dispersive and absorptive parts of the material response must be included when evaluating resonance shifts, modulation depth, and insertion loss.

Below the MIT, the spectral dependence of *n* and κ is determined primarily by interband transitions and, at longer wavelengths, infrared-active lattice vibrations. Above the MIT, mobile carriers add a broad Drude-like contribution that strongly increases infrared absorption and can drive the real part of the permittivity to negative values over part of the infrared spectrum [7,27]. The extinction coefficient generally increases markedly in the near-infrared (NIR), mid-infrared (MIR), and terahertz (THz) regions after metallisation. In contrast, *n* does not necessarily change in a single direction: it can increase or decrease across the MIT, depending on wavelength, because the free-carrier and interband contributions are strongly dispersive.

**Figure 1:**
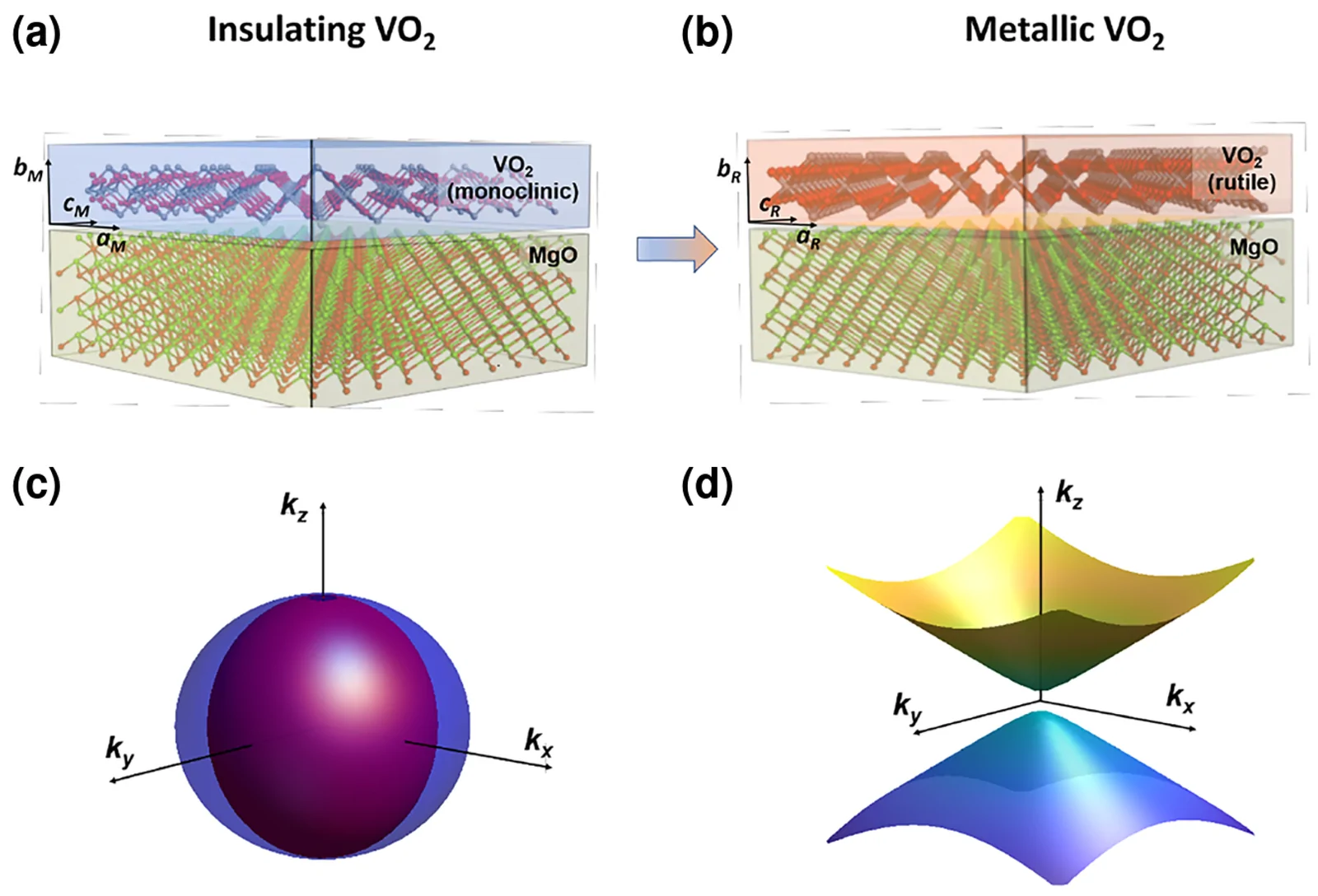
(**a**,**b**) Schematic of the phase-dependent optical anisotropy in epitaxial VO_2_ films on MgO. The epitaxial relationship defines the crystallographic orientation, while the difference between the in-plane and out-of-plane lattice parameters produces birefringence in the insulating phase, as represented by the isofrequency contour in (**c**). During the metal-insulator transition, lattice reconstruction and free-carrier generation modify the dielectric response, producing near-infrared hyperbolic behaviour at λ=1μm, as shown in (**d**). Reproduced from Ref. [14] under the Creative Commons Attribution 4.0 International License.

**Figure 2:**
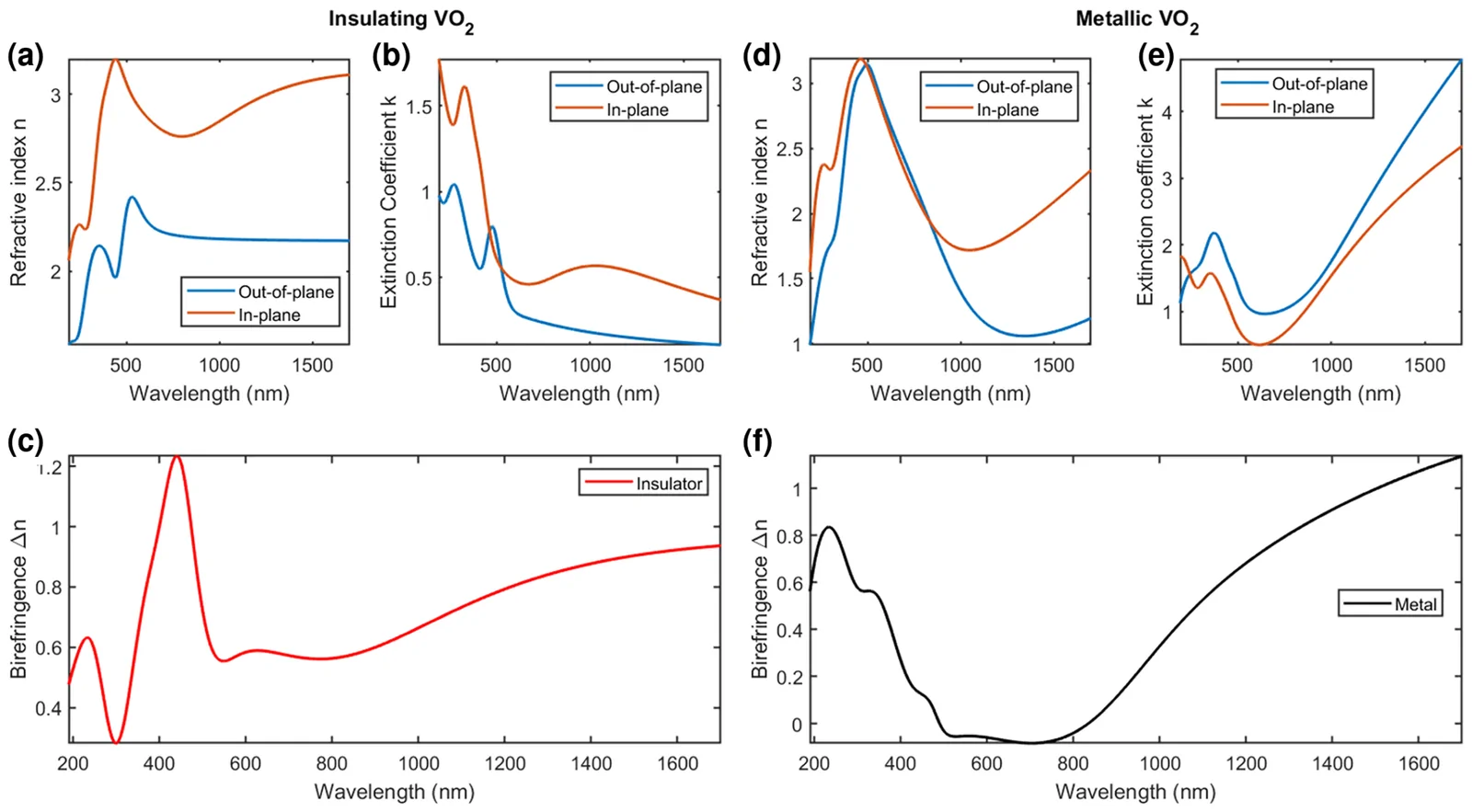
Optical properties of epitaxial VO_2_ thin films obtained by spectroscopic ellipsometry over the 190–1700 nm wavelength range. Panels (**a**,**d**) show the refractive index *n*, panels (**b**,**e**) show the extinction coefficient κ, and panels (**c**,**f**) present the corresponding birefringence Δn in the monoclinic insulating and rutile metallic phases, respectively. Reproduced from Ref. [14] under the Creative Commons Attribution 4.0 International License.

Temperature-dependent ellipsometry from 0.4 to 15 μm has directly resolved the hysteresis of the complex dielectric function during both heating and cooling [25]. Within the coexistence region, *n* and κ evolve with the metallic-domain fraction and topology. Effective-medium models can capture this evolution in some spectral intervals, but their applicability is not universal: for example, a Bruggeman description reproduced visible and NIR data reasonably well in one study but became inaccurate at longer infrared wavelengths [25], whereas THz measurements on another film were better represented by a Maxwell-Garnett treatment coupled to a Drude conductivity [24]. Accordingly, optical constants taken from different publications should not be treated as interchangeable. Film thickness, crystallographic orientation, strain, grain structure, stoichiometry, substrate, and the optical fitting model can all modify the extracted values [13,14]. The influence of crystallographic orientation on the phase-dependent dielectric response is illustrated in Figure 1. The epitaxial constraint produces strong birefringence in insulating VO_2_, while the transition can generate hyperbolic dispersion in the near-infrared [14]. Representative in-plane and out-of-plane optical constants for both phases are compared in Figure 2.

### 2.3. Spectral Dependence from the Visible to the Terahertz

#### 2.3.1. Visible Range

In the visible region, taken here as approximately 0.4–0.7 μm, optical absorption is dominated by transitions involving the O 2p and V 3d bands. The principal high-energy absorption features remain present in both phases, although their amplitudes and spectral positions are modified by the transition [7]. The visible contrast is therefore generally weaker than the infrared contrast, which is advantageous for thermochromic-window concepts that seek to modulate solar infrared transmission while maintaining visible transparency. Nevertheless, VO_2_ remains appreciably lossy in the visible, and interference, film thickness, surface roughness, and additional dielectric layers strongly influence its perceived colour and luminous transmittance.

#### 2.3.2. Near-Infrared Range

The NIR range, approximately 0.7–2.5 μm, contains some of the largest changes relevant to integrated photonics and solar-energy control. In insulating VO_2_, optical features near 0.85 and 1.3 eV arise from transitions between V-derived electronic states. In metallic VO_2_, broadband free-carrier absorption appears below approximately 2 eV [7]. Heating through the MIT therefore commonly decreases NIR transmission and increases reflection and absorption. The resulting contrast is useful at telecommunications wavelengths, including 1.31 and 1.55 μm, but the accompanying increase in κ creates a fundamental trade-off between modulation depth and insertion loss.

#### 2.3.3. Mid-Infrared Range

For wavelengths of approximately 2.5–25 μm, the insulating phase has a relatively weak free-carrier contribution, while optical phonons and interband-transition tails shape the dielectric response. Metallisation produces a strong low-frequency carrier response, causing large changes in reflectance, absorptance, and thermal emittance [25,27]. Depending on wavelength and film quality, metallic VO_2_ can exhibit a negative real permittivity and support plasmonic or hybrid resonances. The MIR is consequently a particularly favourable range for tunable absorbers, thermal emitters, radiative-cooling elements, and reconfigurable metasurfaces. Phonon resonances of VO_2_ and polar substrates must, however, be included in device models because a simple frequency-independent metallic permittivity is inadequate.

#### 2.3.4. Terahertz Range

At frequencies of roughly 0.1–10 THz, corresponding to free-space wavelengths from approximately 3 mm to 30 μm, the response is dominated by low-frequency conductivity and, toward the upper end of the range, lattice vibrations. Insulating VO_2_ is comparatively weakly conducting and can be relatively transparent when sufficiently thin. Across the MIT, the rapid growth of the carrier conductivity strongly suppresses transmission and increases reflection, enabling high-contrast THz switches, modulators, and absorbers [24]. Because THz wavelengths are much larger than typical VO_2_ domains, the transition region is usually described as an effective composite. The resulting response depends not only on metallic volume fraction but also on domain connectivity, which becomes especially important close to the percolation threshold.

The spectral behaviour summarised above demonstrates that the useful optical contrast of VO_2_ and its associated loss arise from the same redistribution of electronic spectral weight. Device optimisation therefore cannot be based solely on maximising the difference between the insulating and metallic optical constants. It must also account for the target wavelength, field overlap with VO_2_, allowed insertion loss, thermal history, and the film-specific dielectric function. These considerations provide the basis for understanding how defects and interfaces modify the performance of VO_2_ nanophotonic structures.

### 2.4. Geometry and Dimensionality

The phase-transition characteristics of VO_2_ depend on geometry because elastic constraints, surface-to-volume ratio, defect density, heat flow, and domain connectivity change between bulk crystals, films, and nanostructures. Bulk and sufficiently thick single crystals provide the closest approximation to an unconstrained transition near 341 K and are valuable for identifying intrinsic crystallographic pathways. Their large thermal mass, however, is poorly matched to low-energy nanophotonic switching.

In epitaxial films, lattice clamping and orientation relative to the substrate determine which rutile-axis strain component is imposed and how the anisotropic dielectric tensor couples to the optical field. Polycrystalline films relax epitaxial strain but introduce grain boundaries, orientation distributions, and spatially nonuniform nucleation thresholds. Ultrathin layers can become dominated by interface chemistry and strain; discontinuous growth, intermixing, or dead layers may weaken the transition, whereas high-quality continuous layers can reduce switched volume and optical overlap without eliminating phase contrast [15,28].

Nanoparticles, nanobeams, nanowires, and patterned resonators introduce free surfaces and finite-size confinement. Single-crystal nanobeams can release substrate clamping and support discrete domains, while nanoparticles embedded in a matrix experience matrix confinement and interface stress. Individual structures may switch through discrete avalanches rather than through the spatially averaged response of a film. These effects can lower switching energy and enable multilevel or stochastic operation, but they also increase sensitivity to individual defects, contacts, and local stoichiometry [29,30]. Dimensionality should therefore be reported together with active dimensions, crystallinity, interface area, substrate coupling, and domain morphology rather than treated as a purely geometric scaling parameter.

## 3. Engineering the VO_2_ Transition

The transition of VO_2_ is highly sensitive to chemical composition, lattice boundary conditions, and microstructure. This sensitivity provides several routes for shifting the metal-insulator transition temperature, $T_{\mathrm{MIT}}$, and tailoring the hysteresis width, transition sharpness, phase contrast, and cycling stability. These quantities are not independent. A modification that lowers $T_{\mathrm{MIT}}$ may also broaden the transition, increase the room-temperature loss, or reduce the difference between the insulating and metallic optical constants. Transition engineering must, therefore, be treated as a multi-parameter optimisation problem rather than as the adjustment of $T_{\mathrm{MIT}}$ alone.

### 3.1. Oxygen Vacancies and Stoichiometry

The narrow stability range of VO_2_ makes oxygen stoichiometry one of the most influential processing variables. Insufficient oxidation can produce oxygen-deficient ${\mathrm{VO}}_{2-\delta }$ or lower vanadium oxides, whereas excessive oxidation can generate V_2_O_5_-rich regions and $V^{5+}$ surface species. Even when secondary phases are not detected by conventional diffraction, small deviations from the ideal V^4+^:O ratio can change the carrier concentration, lattice parameters, and density of transition-nucleation sites.

Defects should be classified by both type and spatial location. Bulk point defects include oxygen vacancies, vanadium vacancies, interstitials, and substitutional dopants. Near-surface nonstoichiometry can differ from the buried active layer because of ambient oxidation, adsorption, or processing damage. Grain boundaries contain under-coordinated atoms, segregated impurities, strain fields, and fast diffusion paths, while heterointerfaces can add charge transfer, band bending, intermixing, misfit dislocations, and oxygen exchange. These populations are not equivalent: a surface $V^{5+}$ component may have little influence on a thick buried channel, whereas a low areal density of interface defects can dominate an ultrathin optical layer.

The commonly reported $V^{3+}$/V^4+^/$V^{5+}$ fractions are useful chemical descriptors but are not unique defect labels. $V^{3+}$-rich character is often consistent with reduction and electron donation associated with oxygen loss; $V^{5+}$-rich character can indicate overoxidation, vanadium vacancies, or V_2_O_5_-like surface coordination; and mixed valence can arise from final-state screening, hydroxylation, beam exposure, or spatial averaging over several phases. The functional consequence depends on location and compensation: electron-rich defects may lower the insulating resistance and transition threshold, but they can also weaken V-V dimerisation, increase cold-state optical absorption, broaden the phase distribution, and accelerate long-term chemical drift.

An oxygen vacancy leaves electrons in the vanadium-oxide lattice and changes the local V-O coordination and V-V bonding. Controlled vacancy creation can therefore reduce the insulating-state resistance and promote metallic behaviour. Experiments on epitaxial films have shown a progressive evolution from a sharp transition to a vacancy-stabilised metallic state as oxygen is removed [13]. Nanoscale control of oxygen defects similarly demonstrated that sufficiently strong reduction can suppress the insulating phase [12]. For photonic devices, moderate vacancy concentrations may lower the switching temperature or create intermediate optical states, but excessive vacancy formation reduces the cold-hot optical contrast and raises the insertion loss because the nominally insulating phase becomes more conductive.

The spatial distribution of oxygen is as important as its average concentration. Surfaces and grain boundaries can overoxidise or act as rapid diffusion paths, while reducing treatments can produce depth-dependent vacancy profiles. Such non-uniformity creates a distribution of local transition temperatures and broadens the macroscopic hysteresis. Conversely, deliberately patterned defect concentrations can define regions that switch at different thresholds, as demonstrated in defect-engineered VO_2_ optical metasurfaces [8]. Reliable stoichiometry assessment should, therefore, combine structural and chemical probes with temperature-dependent electrical or optical measurements; a surface-sensitive oxidation-state measurement alone may not represent the buried active volume.

Vacancy-mediated changes can be reversible or irreversible depending on the pathway. Ordinary thermal cycling through a stoichiometric MIT does not require oxygen removal. By contrast, vacancy creation by high-temperature vacuum annealing, reactive electrodes, or electrolyte gating changes composition and is reversible only if oxygen can be reincorporated or vacancies can back-migrate without secondary-phase formation, roughening, or cation redistribution. Thermal reversibility is therefore generally high while the film remains compositionally stable; chemically induced reversibility is conditional on oxygen chemical potential, diffusion length, temperature, and encapsulation. The broader relevance of defect-controlled electrochemical oxidation in valve-metal oxides is also illustrated by anodically formed, spatially nonuniform niobium-oxide arrays [31], although those results should not be interpreted as direct evidence for a VO_2_ vacancy mechanism.

No single characterisation method uniquely determines oxygen-vacancy concentration or its functional role. Table 2 summarises the complementary information and principal limitations of commonly used probes. Quantitative assignments should therefore combine depth-sensitive chemistry, local structure, spatially resolved microscopy, and temperature-dependent transport or optical measurements on the same specimen.

### 3.2. Epitaxial Strain and Crystallographic Orientation

Strain directly perturbs the V-V chains along the rutile $c_{R}$ axis and modifies the relative free energies of the insulating and metallic phases. The sign and direction of the strain are consequently critical. A representative study of epitaxial VO_2_ on TiO_2_ found that compression of $c_{R}$ reduced $T_{\mathrm{MIT}}$ to approximately 300 K, whereas expansion increased it to approximately 369 K [32]. Strain control through a RuO_2_ buffer layer has produced a continuous transition-temperature range of approximately 285–345 K in ultrathin VO_2_ [33]. In free-standing single-crystal beams, externally imposed strain can also organise alternating metallic and insulating domains and move the transition toward room temperature [29].

Crystallographic orientation determines which lattice direction is clamped by the substrate and which component of the anisotropic dielectric tensor interacts with the optical field. Epitaxial VO_2_ films can therefore show orientation-dependent $T_{\mathrm{MIT}}$, domain morphology, and polarisation response. Temperature-dependent ellipsometry has directly demonstrated tunable optical anisotropy in oriented films [14]. This feature can be useful for polarisation-selective resonators, but it also means that optical constants measured on one orientation cannot automatically be transferred to another.

Nominal lattice mismatch is not sufficient to predict device behaviour. Strain relaxes through misfit dislocations, cracking, grain boundaries, surface roughness, and increasing film thickness. Thermal-expansion mismatch adds a further stress during heating and cooling. The resulting strain distribution can stabilise intermediate monoclinic phases or produce multiple transition steps. Substrate orientation, buffer-layer thickness, VO_2_ thickness, and post-deposition annealing must therefore be considered together, and the residual strain should be measured rather than inferred solely from bulk lattice constants.

### 3.3. W, Mo, Nb, and Other Dopants

Substitutional doping changes both the electronic filling and the lattice. High-valence ions such as W^6+^, Mo^6+^, and Nb^5+^ commonly act as electron donors when they replace V^4+^ and tend to destabilise the insulating state. W is among the most effective dopants for lowering $T_{\mathrm{MIT}}$. In an atomic layer deposition (ALD) film series, increasing the W concentration reduced both the transition temperature and hysteresis; 1.63 at.% W produced a transition near 32 °C [34]. However, the same series showed a smaller change in the temperature-dependent optical constants and emissivity at higher W content. This illustrates the central doping trade-off: a room-temperature transition can be obtained at the cost of reduced phase contrast and a less abrupt response.

Mo doping follows a similar qualitative trend but need not reproduce the W response quantitatively. Mid-infrared ellipsometry of 0–2% Mo-doped films found that $T_{\mathrm{MIT}}$ decreased with dopant concentration while the transition became more gradual; the extracted conductivity also required a Drude-Smith rather than a simple Drude description because of carrier backscattering and film granularity [35]. Nb^5+^ also donates electrons and introduces substantial local lattice distortion because of its larger ionic size. Nb-doped films have shown reduced transition temperature, resistance, and hysteresis, but high dopant levels can widen the overall transition interval and alter the film morphology [36]. Thus, the apparent effect attributed to the dopant may include simultaneous changes in strain, grain size, and phase purity.

Dopants with lower valence or different ionic size can have the opposite effect or stabilise additional insulating phases. Cr^3+^, for example, stabilises the monoclinic M2 and triclinic regions of the VO_2_ phase diagram rather than merely translating a single M1-R transition [22]. Ti, Al, Ga, Fe, F, and hydrogen have also been used to adjust transition behaviour, optical transmission, or environmental stability. Co-doping can partially decouple competing objectives, such as combining a strong reduction in $T_{\mathrm{MIT}}$ with improved visible transmission. Nevertheless, dopant solubility, segregation, compensating oxygen defects, and spatial uniformity must be verified. Dopant concentration alone is not an adequate descriptor of the resulting optical material.

The nominal dopant valence is therefore only a starting point. Ionic radius, site occupancy, compensating defects, clustering, oxygen activity, preferred orientation, and grain size can change simultaneously with composition. A universal transition-temperature shift per atomic percent should not be assumed across growth methods. Table 3 compares the dominant mechanisms and principal optical or stability trade-offs without treating concentration-dependent literature values as directly interchangeable.

### 3.4. Interfaces, Substrates, and Encapsulation

The substrate controls nucleation, texture, epitaxial strain, grain structure, and heat flow. Sapphire and rutile TiO_2_ can support oriented or epitaxial VO_2_, while amorphous SiO_2_ and oxidised Si more commonly produce polycrystalline films unless an appropriate seed or buffer layer is introduced. Comparative growth on sapphire and Si has shown a sharper and larger transition for the epitaxial film, whereas polycrystallinity and a higher defect density weakened the transition on Si [15]. Buffer layers can improve phase selectivity and orientation, block interdiffusion, or impose a designed strain state; their surface roughness and chemical activity are therefore functional parameters rather than passive fabrication details.

Metal contacts are likewise active interfaces rather than electrically neutral terminals. Their work function, chemical reactivity, and deposition damage determine band bending, contact resistance, and local carrier density. Near-surface nonstoichiometry changes the VO_2_ work function across the MIT [37], while sufficiently thin low-work-function layers can donate charge and modify the local transition threshold [38]. In electrically driven devices, contact resistance and current crowding can generate local hot spots that dominate phase nucleation and promote filamentary switching. Interdiffusion, contact oxidation, and stress from thermal-expansion mismatch can progressively shift the threshold during cycling.

Dielectric interfaces control chemistry, electrostatics, optics, and heat flow simultaneously. Band alignment and fixed interfacial charge influence carrier injection; roughness and intermixing produce nonuniform nucleation barriers and optical scattering; and capping layers can either block ambient oxidation or exchange oxygen with VO_2_. Recent layer-selective spectroscopy of VO_2_/W:VO_2_ bilayers showed that sufficiently thin coupled layers can undergo a collective interface-induced transition governed by competition between interfacial and bulk free energies [39]. This result demonstrates that an interface can modify the phase pathway itself rather than merely shift $T_{\mathrm{MIT}}$ through strain.

Interfaces can also exchange oxygen or charge with VO_2_. In TiO_2_/VO_2_ heterostructures, oxygen vacancies created in the TiO_2_ capping layer migrated into VO_2_, lowering the insulating resistance and eventually suppressing the transition [40]. This result shows that a nominally protective cap can modify the active material chemically. Electrode and dielectric deposition conditions must likewise be selected to avoid uncontrolled reduction or oxidation. At the same time, controlled interfacial oxygen transfer offers a route to reversible ionotronic tuning and spatially programmed transition thresholds.

Thermal interfaces determine the energy and time required for switching. The substrate thermal conductivity and VO_2_/substrate thermal boundary resistance set the rate of heat delivery and removal, the cooling time, and the thermal crosstalk between neighbouring elements. Direct thermophysical measurements have shown that the VO_2_ thermal conductivity and the interface heat transmission themselves change across the transition [41]. Consequently, simulations that use a temperature-independent thermal stack can misestimate the threshold power and recovery dynamics.

Encapsulation is essential when long-term exposure to oxygen, moisture, or elevated temperature is expected. VO_2_ can gradually overoxidise and lose its thermochromic contrast, particularly through porous surfaces and exposed film edges. Full HfO_2_ encapsulation of both the surface and cross-sections substantially improved stability under accelerated high-temperature and high-humidity testing while also functioning as an optical antireflection layer [42]. A cap layer must nevertheless be evaluated for added stress, oxygen exchange, optical interference, parasitic absorption, and thermal resistance. The most chemically inert coating is not necessarily the optimum photonic or thermal interface.

Interface qualification should extend beyond initial performance. Relevant failure modes include contact oxidation, oxygen redistribution, interdiffusion, vacancy trapping, roughening, cracking, delamination, and cap-layer densification. Long-term tests should monitor contact resistance, interfacial chemistry and roughness, transition threshold, optical contrast, and write/reset dynamics after thermal, electrical, and environmental cycling. A stable crystallographic phase alone does not demonstrate stable device interfaces.

### 3.5. Film Thickness, Grain Size, and Dimensionality

Reducing the VO_2_ thickness lowers the active volume, thermal mass, and, in many geometries, optical insertion loss. However, ultrathin films are more strongly influenced by the substrate interface and may become discontinuous, poorly crystallised, or chemically non-uniform. Thickness also controls strain relaxation. In VO_2_/Al-doped-ZnO heterostructures, changing the VO_2_ thickness and interface roughness modified the residual strain and continuously shifted the transition temperature [43]. The useful minimum thickness is therefore process-dependent rather than a universal material constant.

Grain size controls both phase nucleation and carrier transport. Grain boundaries supply defects and potential nucleation sites, so smaller grains can reduce $T_{\mathrm{MIT}}$ and modify the hysteresis. For example, an undoped film with an approximately 30 nm grain size exhibited a transition near 45 °C [44]. However, grain refinement can also increase boundary scattering, oxygen non-uniformity, and metallic-state resistance. Moreover, annealing commonly changes grain size and crystallinity simultaneously. The observed hysteresis therefore results from competing effects: larger volumes are statistically more likely to contain a nucleation site, while higher crystalline perfection can remove the defects that facilitate nucleation. Size-dependent studies of isolated VO_2_ precipitates consequently report systematic changes in both transition temperature and hysteresis [45].

Changing dimensionality from continuous films to nanoparticles, nanobeams, or nanowires further alters elastic boundary conditions and phase-domain dynamics. Free-standing nanostructures can partly release substrate clamping, whereas embedded particles experience matrix confinement and interfacial stress. Individual structures may switch through discrete domain events rather than the spatially averaged transition observed in a film. Solution-grown single-crystalline VO_2_ nanostructures have exhibited transition temperatures as low as 32 °C, although dimensional scaling and stoichiometry were both implicated [30]. Dimensionality can thus lower switching energy and enable discrete or multilevel states, but it may also increase device-to-device variability because the response becomes sensitive to individual defects and contacts.

These engineering parameters are strongly coupled. Oxygen activity during growth influences vacancy density and grain size; dopants modify both carrier concentration and strain; thickness determines strain relaxation and interface dominance; and encapsulation can affect oxygen chemical potential, stress, optics, and heat flow simultaneously. Meaningful comparison between VO_2_ devices therefore requires reporting not only $T_{\mathrm{MIT}}$, but also the heating and cooling branches, hysteresis width, transition slope, optical constants or modulation contrast, phase purity, thickness, orientation, grain structure, stoichiometry, substrate stack, and cycling history. Such multidimensional characterisation is necessary to translate transition engineering into reproducible nanophotonic performance.

## 4. Activation Mechanisms

The metal-insulator transition of VO_2_ can be activated by heat, light, electrical bias, mobile ions, or mechanical boundary conditions. Although these stimuli may produce similar changes in resistance or optical transmission, they do not necessarily follow the same microscopic pathway or reach the same final state. The activation mechanism determines the switching speed, energy consumption, spatial selectivity, volatility, endurance, and degree of compatibility with an integrated photonic platform. In particular, thermally, optically, and electrically driven responses are often coupled: absorbed photons or electrical current can heat the lattice above $T_{\mathrm{MIT}}$ even when the applied stimulus is not itself thermal. Mechanism identification therefore requires more than observing an abrupt optical or electrical change.

### 4.1. Thermal Switching

Thermal activation is the most direct and experimentally reproducible way to switch VO_2_. A global temperature stage, a resistive heater beneath or beside the active region, or heat delivered through the substrate raises the local temperature across the equilibrium transition. The metallic phase persists while the device remains above the heating branch of the hysteresis and reverts to the insulating phase after cooling below the corresponding cooling branch. Ordinary thermal switching is therefore volatile, although the hysteresis can support two possible states over a limited temperature interval. The actual state within this interval depends on the previous thermal trajectory and cannot be specified by temperature alone.

The thermal response of a device can be approximated by a lumped time constant

(3) $$\tau_{\mathrm{th}}≃R_{\mathrm{th}}C_{\mathrm{th}},$$

where $R_{\mathrm{th}}$ is the effective thermal resistance between the active region and its surroundings, and $C_{\mathrm{th}}$ is the thermal capacitance of the heated volume. Reducing the VO_2_ volume and heater footprint lowers the sensible heat required for switching, while a low-resistance thermal path accelerates cooling. These objectives compete: strong thermal isolation reduces the holding power but increases the recovery time and thermal crosstalk. The latent heat and temperature-dependent thermophysical properties must also be included close to the transition. Measurements have shown that both the VO_2_ thermal conductivity and the VO_2_/substrate heat transmission change across the hysteresis [41].

Global heating provides a relatively uniform phase fraction and is useful for material characterisation, but it is generally too slow and energy intensive for pixel-level reconfiguration. Integrated microheaters confine the thermal stimulus and can independently address resonators or metasurface elements. Their performance depends on heater resistance, dielectric isolation, electrode geometry, substrate thermal conductivity, and the distance between the heater and VO_2_. Temperature gradients may cause sequential domain nucleation rather than uniform switching, producing a gradual optical response even when the intrinsic film transition is sharp. Thermal devices should therefore report the local or simulated active-region temperature, heating and cooling times separately, duty cycle, steady-state holding power, and crosstalk to adjacent elements.

### 4.2. Optical Excitation

Optical activation occurs in two limiting regimes. Under continuous-wave illumination or pulses that deposit energy more slowly than electron-phonon thermalisation, absorption in VO_2_ or an adjacent resonator raises the lattice temperature. This photothermal pathway follows the equilibrium hysteresis and is governed by the absorbed, rather than incident, optical power. Resonant field enhancement can reduce the external threshold by concentrating absorption in a small volume, while the phase-induced increase in absorption can provide positive feedback and an abrupt nonlinear response. The resulting recovery is limited by heat removal and is usually much slower than the initial electronic response.

**Figure 3:**
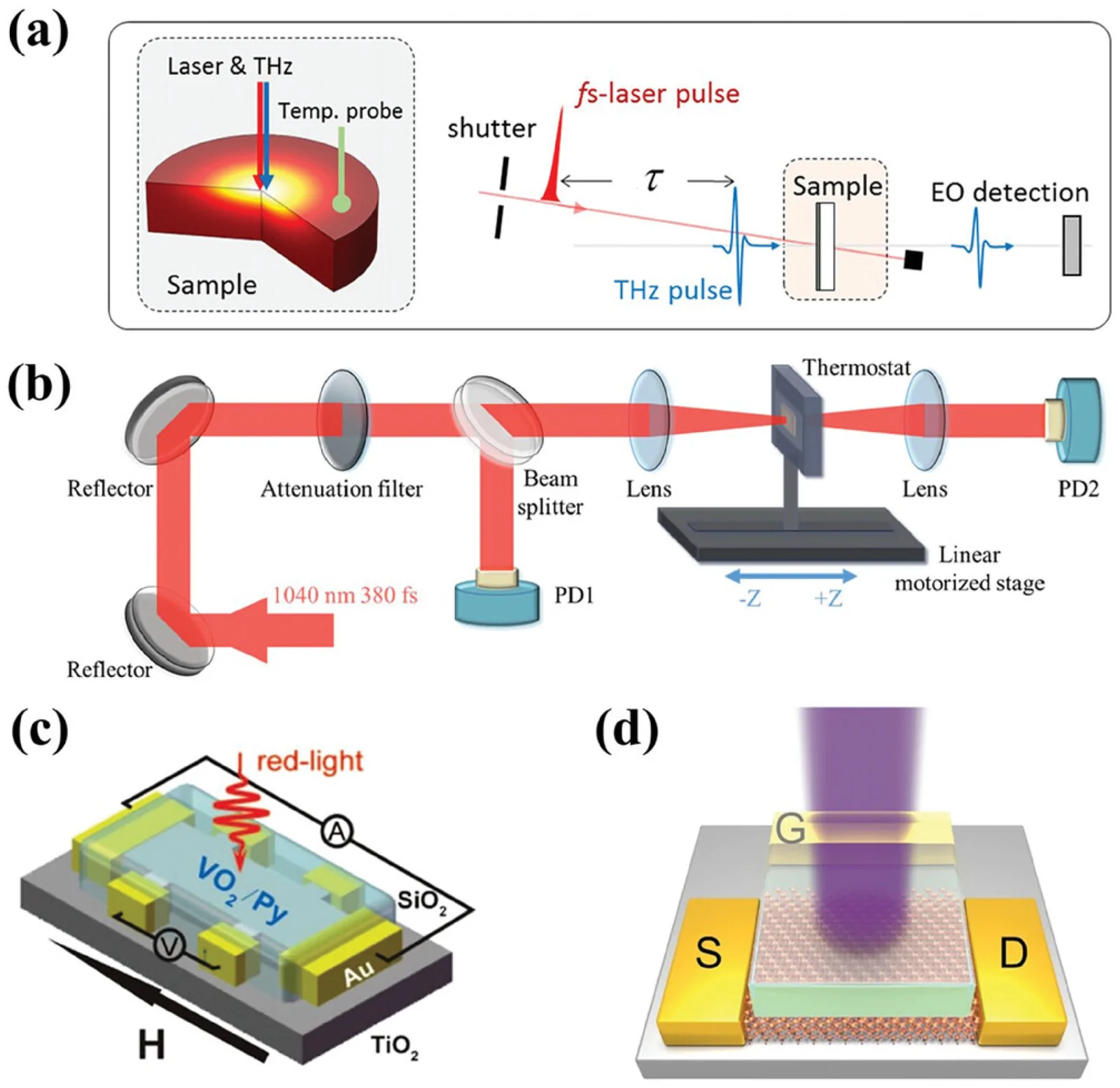
Representative approaches for optically activating and characterising the VO_2_ metal-insulator transition. (**a**) Kinetic terahertz absorption (KITA) configuration used to probe the accumulated photothermal response during pulsed excitation. Adapted with permission from Ref. [46]. © 2018 Optical Society of America. (**b**) Variable-temperature Z-scan arrangement used to characterise the nonlinear optical response. Adapted with permission from Ref. [47]. © 2022 Optica Publishing Group. (**c**) Phase-change anisotropic magnetoresistance (PCAMR) device used to investigate optically controlled transport in a NiFe/VO_2_ heterostructure. Adapted with permission from Ref. [48]. © 2020 Wiley-VCH Verlag GmbH & Co. KGaA. (**d**) VO_2_ neuromorphic transistor activated by 375 nm ultraviolet illumination, in which the VO_2_ channel connects the source and drain electrodes and an ionic liquid provides gate control. Adapted from Ref. [49] under the Creative Commons Attribution 4.0 International License.

Representative configurations for photothermal excitation, nonlinear optical characterisation, and optoelectronic control of VO_2_ are summarised in Figure 3. These architectures illustrate how the optical stimulus, thermal environment, and device geometry determine the observed switching response.

Intense femtosecond excitation can perturb the electronic population and lattice potential before an equilibrium temperature is established. In this regime, “the transition” comprises several observables that need not evolve together. Carrier redistribution and optical-gap collapse can occur first, followed by loss of V-V dimer and zigzag order and finally by the macroscopic development of tetragonal lattice symmetry. Femtosecond X-ray measurements observed a sub-picosecond transformation at sufficiently high excitation density [50], while metal-like optical responses have been reported in a monoclinically distorted lattice [51]. Mega-electron-volt ultrafast electron diffraction found that dimer and zigzag order disappeared within approximately 200 fs and tetragonal symmetry developed over approximately 5 ps [26]. The data therefore support strong coupling between electronic and structural degrees of freedom without requiring strict simultaneity far from equilibrium.

A fast pump-probe signal should consequently not be interpreted automatically as complete conversion to the equilibrium rutile metal. Whether a distinct monoclinic metallic state exists, and how long it persists, depends on fluence, initial temperature, strain, microstructure, repetition rate, and probe sensitivity. Sub-threshold excitation may create short-lived carriers without a phase transition, whereas repeated pulses can accumulate heat and produce an effectively photothermal response. A rigorous optical-switching study should state the pump wavelength, pulse duration, spot size, absorbed fluence, repetition rate, initial temperature, and recovery criterion, and should distinguish an electronic response from a structurally confirmed rutile conversion.

### 4.3. Electrical and Joule-Heating Activation

Electrical switching is attractive because the control signal can be generated and routed on-chip. Current can pass directly through the VO_2_ channel or through a separate metal heater. In both cases, the dissipated power,

(4) $$P_{J}=IV=I^{2}R=\frac{V^{2}}{R},$$

raises the local temperature. In a direct two-terminal device, an initially warmer or more conductive region carries additional current, nucleates the metallic phase, and can develop into a conducting filament. The associated electrothermal feedback produces threshold switching, an abrupt resistance collapse, and, under suitable circuit conditions, negative differential resistance.

Spatially resolved thermometry has shown that the local temperature reaches the thermal transition during DC voltage- or current-induced switching, establishing Joule heating as the predominant mechanism in those devices [52]. Correlated maps of temperature and structure have likewise shown that conductance switching coincides with redistribution of the thermal profile and the appearance of a rutile filament [53]. These findings do not exclude field-assisted carrier injection or nonequilibrium electronic effects in very short, nanoscale devices, but they show that a threshold voltage alone is not evidence for a purely electric-field-driven transition. Distinguishing the mechanisms requires local thermometry, pulse-width and ambient-temperature scaling, and preferably a simultaneous structural probe.

Passing current through a neighbouring heater separates the electrical control path from the VO_2_ resistance and can improve uniformity, current compliance, and endurance. For example, a VO_2_-metal metasurface in which the patterned metal provided Joule heating demonstrated an electrically controlled mid-infrared transmission ratio of approximately 100 [54]. Direct current injection can require a smaller footprint, but contact resistance, current crowding, filament localisation, and electromigration complicate scaling. In both configurations, the turn-off time is often limited by cooling rather than by the intrinsic electronic transition. Recent operando electron microscopy under MHz–GHz electrical excitation directly resolved domain nucleation and a structural-phase-front velocity of 4.54 nm ns^−1^; incomplete structural recovery limited reversible cycling at high frequency [55]. Electrical switching metrics should therefore include the drive circuit, pulse shape, compliance, duty cycle, active temperature, turn-on and recovery times, and energy delivered to the complete heater stack.

### 4.4. Electrochemical and Ionic Control

Field-effect control of VO_2_ can follow several physically distinct pathways. Pure electrostatic gating changes interfacial carrier density within the screening length without intentionally changing stoichiometry. Its response should be volatile when the bias is removed and can, in principle, be fast, although the measured speed is limited by gate resistance and capacitance. Because electronic screening is short in correlated oxides, a large persistent change through a comparatively thick film is unlikely to be purely electrostatic.

Electrochemical oxygen-vacancy formation or migration changes the ${\mathrm{VO}}_{2-x}$ composition and redistributes $V^{3+}$/V^4+^ character. Ionic-liquid gating has removed oxygen from epitaxial VO_2_, created oxygen vacancies, and stabilised a metallic state even after removal of the electrolyte [56]. Such switching is nonvolatile but reaction- and diffusion-limited, and reversal requires controlled reoxidation or vacancy back-migration. The original insulating state may not be fully recovered if cycling causes electrolyte decomposition, surface roughening, or secondary phases.

Proton insertion is a second composition-changing pathway. Interstitial hydrogen donates charge and expands the lattice; moderate insertion can stabilise a conducting phase, whereas stronger insertion can create a second insulating hydrogen-rich phase. Reversible electrolyte-gating-induced protonation has been demonstrated experimentally [57]. With a solid-state proton conductor, low gate bias produced an insulator-to-metal response, whereas stronger insertion generated an insulating hydrogen-rich phase, giving an insulator-metal-insulator sequence and approximately 7% out-of-plane chemical expansion [58]. Other mobile ions, including Li^+^ or Na^+^, can similarly intercalate and modify both structure and electronic occupancy. “Electrochemical reduction” is therefore an umbrella description of a redox process, whereas vacancy migration, protonation, and cation intercalation identify the mobile species and resulting composition.

Mechanism assignment requires more than a gate-voltage-dependent resistance. Volatility after removing the bias, polarity dependence, thickness scaling, temperature-activated kinetics, isotope tracing, mass or composition changes, lattice expansion, and depth-resolved spectroscopy should be evaluated. For photonic applications, the relevant endurance criterion is recovery of the complex optical constants and surface/interface quality, not conductance alone. Table 4 compares the expected retention, speed, and reversibility limits of the principal pathways.

A reversible gate sweep or repeated conductance trace should not be presented as endurance unless the test reports the number of complete write–reset cycles, pulse amplitude and duration, duty cycle, temperature, read condition, failure criterion, and recovery of the optical response. The absence of a transferable cycle count in Table 4 therefore reflects the lack of standardised cross-mechanism testing rather than an assumption of poor endurance.

### 4.5. Strain and Ferroelectric Control

Mechanical activation exploits the strong coupling between the VO_2_ electronic state and the V-V chain geometry. Uniaxial tension or compression can shift the relative stability of insulating and metallic phases, move domain walls, and change the transition threshold. In free-standing single-crystal beams, applied strain organises alternating metallic and insulating domains and permits continuous control of their fractions [29]. Mechanical bending or a microelectromechanical actuator can thus modulate the optical response without passing current through VO_2_, although the actuator size and mechanical resonance may limit integration and speed.

Piezoelectric and ferroelectric substrates convert an applied voltage into in-plane strain through the converse piezoelectric effect. Because VO_2_ is mechanically coupled to the substrate, this strain changes its transition temperature or resistance. In VO_*x*_/PMN-PT heterostructures, ferroelastic domain switching generated two remanent strain states, shifted the transition temperature by as much as 6 K, and changed the resistance by up to 40% near the transition [59]. Unlike an ordinary linear piezoelectric response, a remanent ferroelastic state can retain the programmed VO_2_ response after the control pulse is removed.

Ferroelectric polarisation may also act through interfacial charge and orbital coupling rather than strain alone. A VO_2_/BiFeO_3_/SrRuO_3_ heterostructure demonstrated nonvolatile room-temperature control with a conductance on/off ratio of approximately $10^{2}$, retention of approximately $10^{4}$ s, and endurance of approximately $10^{3}$ cycles [60]. In practice, strain, polarisation charge, defect redistribution, and interfacial chemistry can coexist, and their relative contributions depend strongly on VO_2_ thickness and interface quality. Appropriate control samples and simultaneous strain, polarisation, and composition measurements are, therefore, needed before assigning the response to ferroelectric field effect alone.

No activation mechanism is universally optimal. Thermal actuation is simple and produces an equilibrium phase but consumes holding power; optical excitation provides remote addressing and the fastest initiation but can require high peak fluence; electrical heating is readily integrated but is susceptible to hot spots and thermal crosstalk; ionic control offers nonvolatile multilevel states at the cost of slower chemical dynamics; and strain or ferroelectric control can reduce static power but adds heterostructure and actuator constraints. Meaningful comparison requires reporting the complete write and reset process, including the initial state, stimulus waveform, active volume, absorbed or delivered energy, local temperature, optical modulation, recovery time, retention, and endurance. These quantities reveal whether a proposed device exploits the intrinsic phase-transition dynamics or is limited by heat flow, ion motion, or an external actuator.

## 5. Nanophotonic Applications

VO_2_ is useful in nanophotonics because its transition changes both the real and imaginary parts of the optical permittivity. A single patterned structure can therefore act as a switch, phase shifter, absorber, filter, or thermal emitter, depending on how the electromagnetic mode overlaps the VO_2_ and how the device is excited and observed. These categories are not mutually exclusive: a resonant absorber is also a thermal emitter under equilibrium conditions, and a tunable metasurface can simultaneously modulate amplitude, phase, and polarisation. Application claims should consequently be supported by optical measurements at the operating wavelength rather than by electrical resistance alone. Relevant figures of merit include modulation depth, insertion loss, accessible phase range, spectral bandwidth, switching energy and time, recovery time, aperture or coupling efficiency, endurance, and thermal crosstalk.

### 5.1. Active Metasurfaces and Metamaterials

Metasurfaces use subwavelength resonators to impose a spatially varying complex transmission or reflection coefficient. Incorporating VO_2_ allows the phase transition to alter the resonant current path, effective capacitance and inductance, radiative coupling, and material loss. In hybrid metal-VO_2_ meta-atoms, the metallic pattern usually supplies a strong resonance while VO_2_ opens or closes a conducting path or changes the dielectric environment. Patterned VO_2_ can itself form switchable dielectric resonators, reducing the dependence on metallic elements but requiring tight control of crystal quality, dimensions, and anisotropy.

Early terahertz demonstrations established that electrically controlled VO_2_ domains could provide persistent frequency tuning in a metamaterial response [61], while VO_2_ integrated with nanoscale antennas enabled active control of terahertz resonances [62]. At optical and infrared frequencies, defect engineering has been used to tailor the nucleation and hysteresis of active VO_2_ metasurfaces [8], and epitaxial VO_2_ nanostructures have produced large-area switchable dielectric metasurfaces [10]. Electrically heated devices have demonstrated uniform phase modulation [9] and multifunctional mid-infrared switching and limiting [54], while binary-addressed beam steering has been investigated computationally [11].

The central scaling challenge is local control. Beam steering and wavefront shaping require reproducible phase states across many meta-atoms, ideally approaching a 2π phase range with high and nearly uniform amplitude. In VO_2_, however, neighbouring pixels are coupled by heat diffusion, while grain-to-grain variations broaden the local transition and create nonuniform phase fractions. Resonant enhancement increases the optical effect of a small VO_2_ volume but also narrows the operating bandwidth and makes the response sensitive to fabrication tolerances. Practical active apertures must therefore be evaluated for addressable pixel pitch, phase quantisation, side-lobe level, aperture efficiency, electrical routing, thermal crosstalk, and stability over repeated cycles.

### 5.2. Optical Switches and Modulators

VO_2_ switches and modulators can operate in free space or in a guided mode, and can encode information in intensity, phase, or polarisation. For an intensity device, two commonly reported quantities are the extinction ratio and insertion loss,

(5) $$\mathrm{ER}=10\log_{10}\;\left(\frac{P_{\mathrm{on}}}{P_{\mathrm{off}}}\right),\;\mathrm{IL}=-10\log_{10}\;\left(\frac{P_{\mathrm{on}}}{P_{\mathrm{in}}}\right),$$

where the definition of the transmitting “on” state must be stated explicitly. A large resistance ratio does not guarantee a large ER, because the optical mode may overlap only weakly with VO_2_. Conversely, increasing the overlap enhances modulation but also raises propagation loss in one or both phases.

Resonant modulators amplify a small permittivity change through a cavity or metasurface resonance, permitting compact footprints and potentially lower switching energy. Their disadvantages are a narrower optical bandwidth, temperature-dependent resonance detuning, and sensitivity to fabrication error. Nonresonant waveguide devices offer broader bandwidth but require either a longer interaction length or stronger modal overlap. A silicon waveguide containing a 500-nm-long VO_2_ section demonstrated approximately 10 dB broadband switching contrast [63]. In a silicon waveguide clad with VO_2_, measured propagation losses were 1.27 and 4.55 dB μm^−1^ in the insulating and metallic states, respectively, illustrating both the compactness and the insertion-loss penalty of strong overlap [64].

The intrinsic transition can begin much faster than the thermal response of a complete device. Electrically triggered VO_2_ integrated with an optical resonator has shown turn-on faster than 2 ns [65], but the repetition rate may still be limited by cooling and structural recovery. Reports should therefore separate material-transition time, optical rise and fall times, electrical-pulse duration, and sustainable modulation bandwidth. Energy per transition should include the heater, electrodes, and parasitic capacitance, while a thermally maintained metallic state also requires reporting static holding power.

### 5.3. Tunable Absorbers and Filters

VO_2_ can tune absorption by changing both resonant impedance matching and internal dissipation. A common geometry combines a patterned top layer, a dielectric or VO_2_ spacer, and a reflecting backplane. At critical coupling, radiative leakage is balanced by absorption and reflection is suppressed; switching VO_2_ perturbs this balance and changes the absorption amplitude, linewidth, or centre wavelength. In hybrid gold-VO_2_ resonators, thermal switching changed the resonant absorption from approximately 90% to 20% for a nanowire design and from 96% to 32% for a nanodisc design [66].

The same principles enable tunable spectral filters. A transition-induced resonance shift is useful for wavelength selection, whereas a change in damping can gate an otherwise fixed passband or stopband. Narrow resonances improve spectral selectivity but are vulnerable to roughness, thickness error, and the spatial coexistence of insulating and metallic domains. Broadband absorbers tolerate these variations more readily but provide less spectral discrimination. A meaningful comparison requires angle- and polarisation-resolved spectra over both heating and cooling, because a large normal-incidence contrast at one wavelength may not survive over the numerical aperture or temperature range of an imaging system.

### 5.4. Adaptive Thermal Emitters

For a reciprocal body in local thermal equilibrium, Kirchhoff’s law relates directional spectral emissivity to absorptivity,

(6) ε(λ,θ,ϕ,T)=A(λ,θ,ϕ,T).

The absorbers discussed above can thus become active thermal emitters when heated. This connection is especially useful for VO_2_ because temperature is both the emitted-radiation variable and the switching stimulus. A VO_2_ film on sapphire has exhibited a narrow, nearly blackbody-like emission feature together with broadband negative differential thermal emittance, for which emitted power over part of the spectrum decreases as temperature rises through the transition [67]. Patterned gap-plasmon metasurfaces have inverted the ordinary thermal-radiation contrast of VO_2_ [68], showing that nanophotonic design can determine whether the hot metallic state appears brighter or darker.

**Figure 4:**
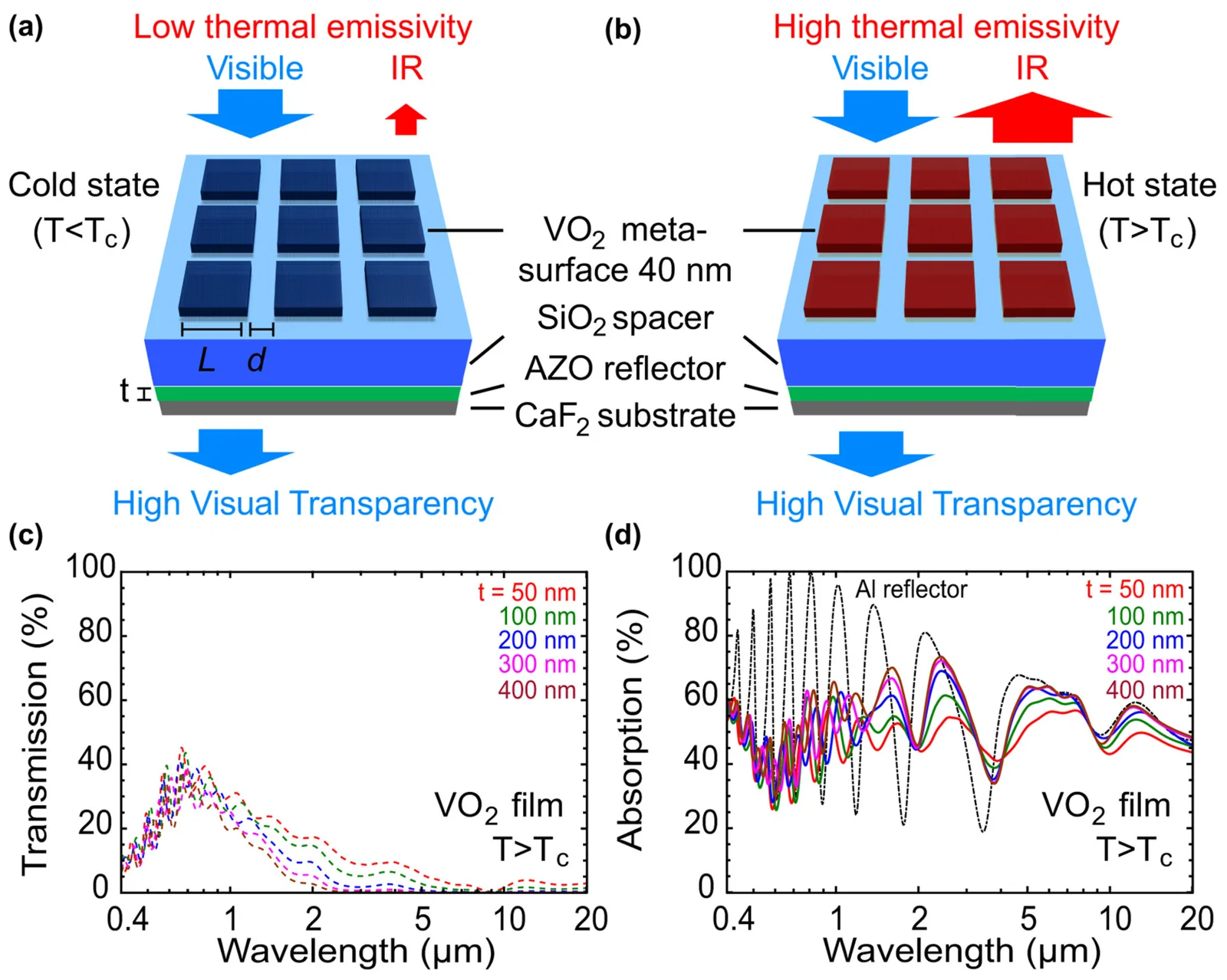
Operating concept of a VO_2_ metasurface thermal emitter and the simulated response of an unpatterned reference stack. Panels (**a**,**b**) illustrate the patterned device in its cold and hot states. Panels (**c**,**d**) show the calculated transmission (dashed curves) and absorption (solid curves), respectively, of the unpatterned VO_2_-film stack in the hot state ($T>T_{C}$), for Al-doped ZnO (AZO) reflector thicknesses of 50–400 nm. The dash-dotted curve in panel (**d**) represents the corresponding Al-reflector stack. Reproduced from Ref. [69] under the Creative Commons Attribution 4.0 International License.

A representative visibly transparent, thermochromic emitter is shown in Figure 4. In this architecture, switching the VO_2_ metasurface modifies the infrared emissivity, while the Al-doped ZnO reflector preserves substantial visible transmission [69].

Spatially selective activation extends this concept from uniform coatings to programmable infrared scenes. A dynamically reconfigurable VO_2_ emitter demonstrated emissivity tuning from 0.19 to 0.91 over the 8–14 μm band and as many as nine distinguishable emission levels [70]. Such structures are relevant to radiative thermal regulation, infrared calibration sources, adaptive signatures, and information display. Device assessment should nevertheless use angle-integrated emittance and net radiative heat flow when thermal management is the target; normal-incidence reflectance alone is insufficient. Oxidation resistance, encapsulation, temperature uniformity, atmospheric-window bandwidth, viewing angle, and cycling stability are equally important for deployment.

### 5.5. Sensing and Imaging

VO_2_ can participate in an imaging system as a detector material, a reconfigurable optical element, or a controllable emitter. In a bolometer, absorbed infrared radiation heats a suspended pixel and changes its resistance. Operation close to the transition can provide a large temperature coefficient of resistance, but it also makes the response sensitive to hysteresis, drift, low-frequency noise, and pixel-to-pixel variation. An early VO_2_ uncooled microbolometer linear array demonstrated eight-element detection in the 8–12 μm band [71]. This phase-transition use should be distinguished from commercial “VO_*x*_” microbolometers that may employ amorphous vanadium oxide as a thermistor without deliberately switching crystalline VO_2_ through its transition.

At the optical front end, a VO_2_ metasurface can switch polarisation, spectral, or spatial response before the radiation reaches a focal-plane array. A far-infrared metasurface operating across 8–14 μm was transparent to both linear polarisations in the insulating state but selectively suppressed one polarisation in the metallic state, producing a linear-polarisation extinction ratio of up to 10 dB and enabling switchable intensity and polarisation imaging [72]. This type of multifunctional aperture can reduce the number of separate filters or polarisers, although nonuniform heating and calibration drift must be controlled across the field of view.

**Figure 5:**
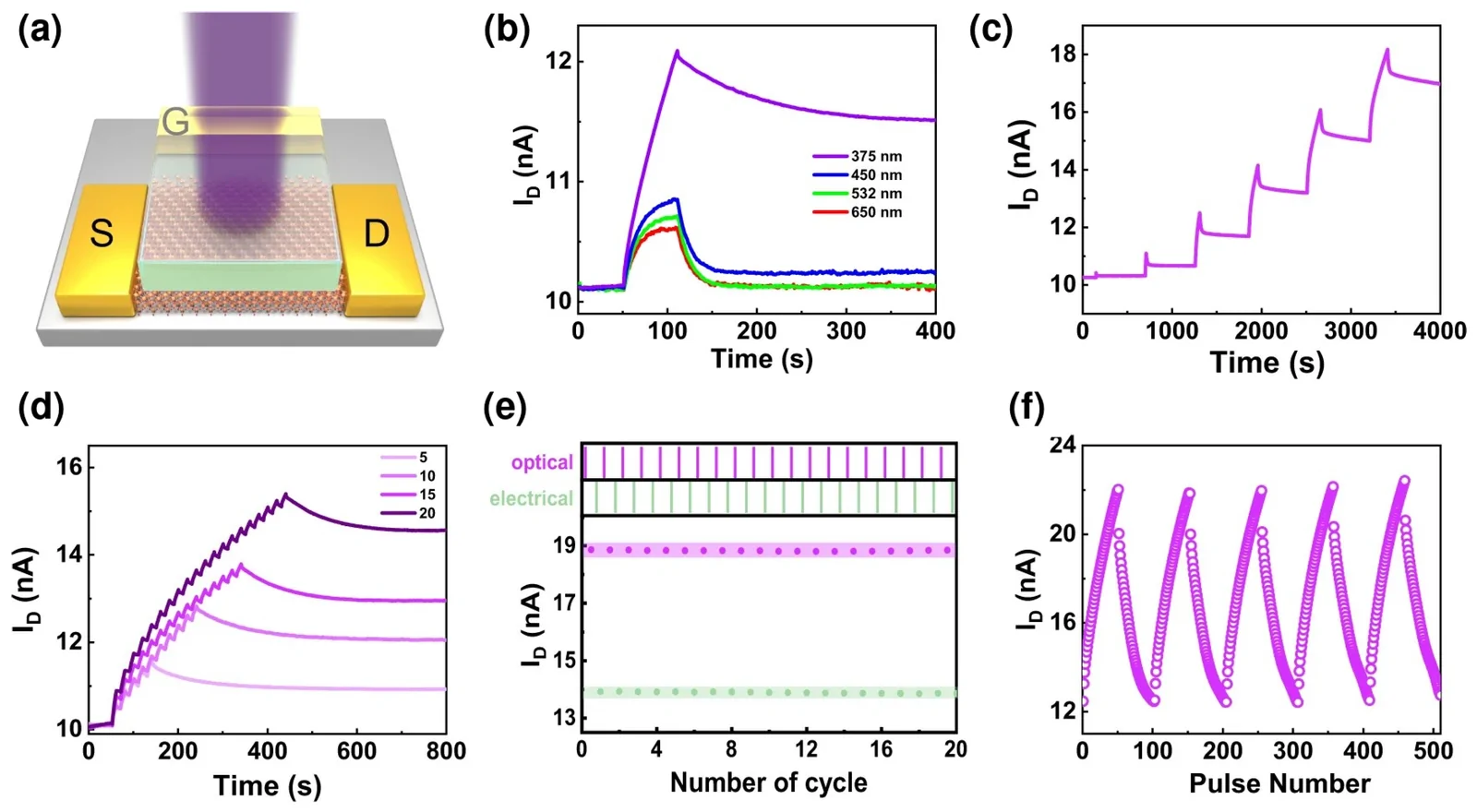
Optoelectronic response and synaptic operation of a VO_2_ neuromorphic transistor. (**a**) Device configuration under 375 nm ultraviolet excitation, with the VO_2_ film forming the channel between the source and drain electrodes and an ionic liquid acting as the gate medium. (**b**) Temporal drain-current response, $I_{D}$, under illumination at different wavelengths for 60 s at 64 mW cm^−2^. (**c**) Dependence of $I_{D}$ on UV-exposure duration at 84 mW cm^−2^. (**d**) Spike-number-dependent plasticity produced by 10 s UV pulses. (**e**) Reversible switching using optical potentiation (84 mW cm^−2^, 20 s) and electrically induced depression (−2.5 V, 20 s); $I_{D}$ was measured 2 s after each stimulus. (**f**) Long-term potentiation induced by UV pulses and long-term depression controlled by gate-voltage pulses ranging from −1.5 to −3.5 V. A constant source-drain voltage of $V_{\mathrm{SD}}=50$ mV was used for channel-current measurements. Reproduced from Ref. [49] under the Creative Commons Attribution 4.0 International License.

Beyond conventional infrared detection, photoinduced nonvolatile switching enables VO_2_ devices to combine sensing, memory, and information processing. As shown in Figure 5, the response of an ionic-gated VO_2_ transistor depends on illumination wavelength, UV dose, and pulse number, enabling optical potentiation and electrically controlled depression [49].

VO_2_ resonators can also support refractive-index, chemical, or thermal sensing. An analyte changes the resonance through its overlap with the near field, while the VO_2_ transition provides an adjustable baseline, a reference state, or a means of moving the resonance across a molecular band. The same increased absorption that produces strong switching can lower the quality factor and degrade the smallest resolvable spectral shift. Sensor studies should therefore report sensitivity together with linewidth, noise, repeatability, hysteresis, and a system-level limit of detection, rather than resonance shift alone.

### 5.6. Integrated Photonic Devices

Integrated platforms concentrate an optical mode into a small VO_2_ volume and provide electrical access, enabling compact switches, modulators, detectors, and nonlinear elements. VO_2_ has been combined with silicon and silicon-on-insulator waveguides, resonators, and plasmonic structures. The modal overlap factor provides the basic design trade-off: placing VO_2_ at a field maximum shortens the required interaction length but increases insertion loss, whereas evanescent or slot coupling offers greater freedom to balance loss and contrast. The waveguide demonstrations discussed above show that sub-micrometre active lengths and approximately 10 dB contrast are feasible, but also that propagation loss can reach several decibels per micrometre when overlap is strong [63,64].

**Figure 6:**
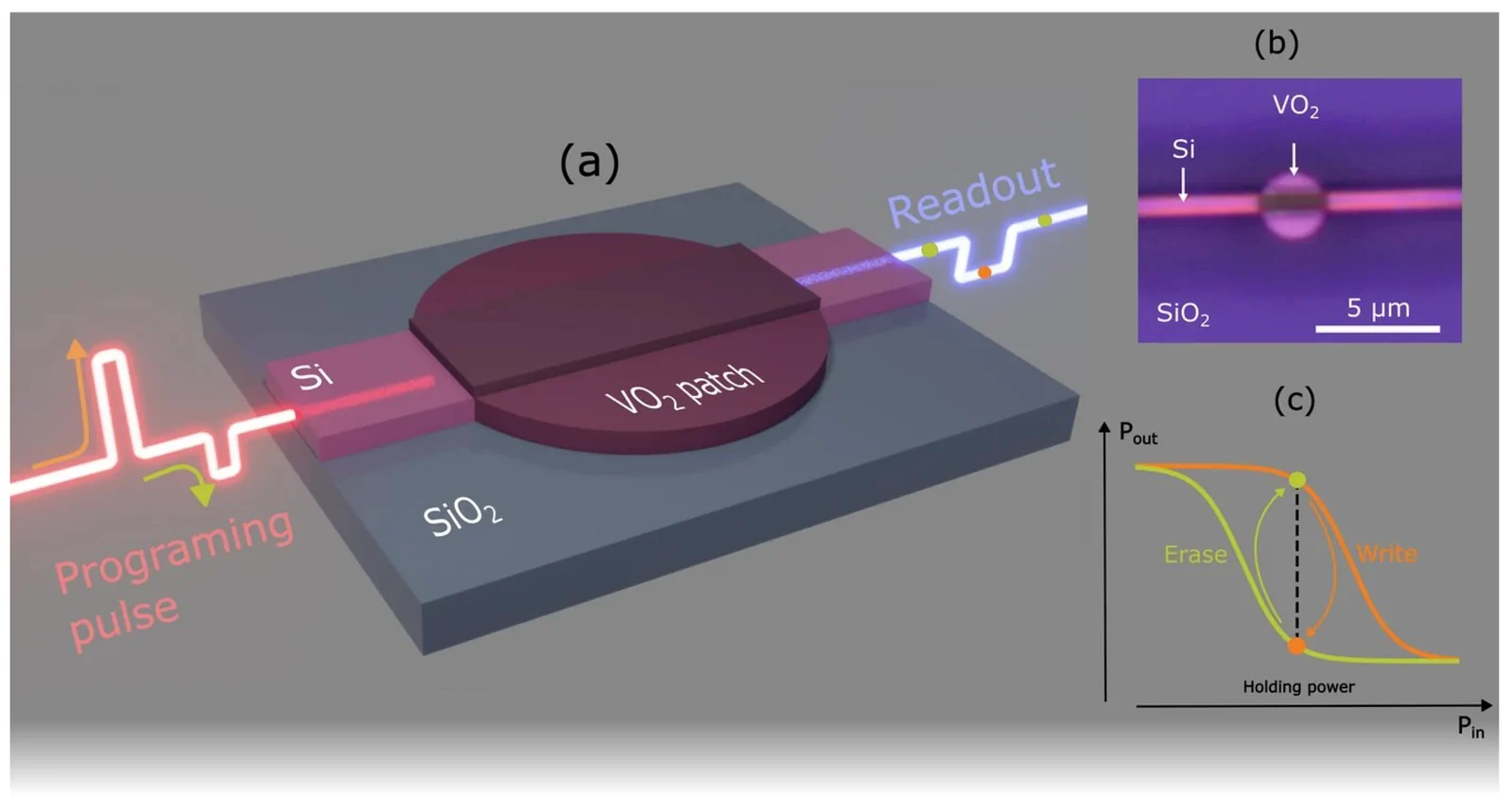
Operating concept and implementation of a VO_2_/Si photonic memory. (**a**) Schematic of the memory programmed and reset using optical write and erase pulses. (**b**) Optical micrograph of the fabricated device. (**c**) Illustration of switching between the written and erased states by exploiting the hysteresis of the VO_2_ metal-insulator transition. In (**c**), the orange arrow indicates writing by increasing the optical input power, whereas the yellow-green arrow indicates erasing by decreasing it. The dashed vertical line marks the holding-power level; $P_{\mathrm{in}}$ and $P_{\mathrm{out}}$ denote the input and output optical powers, respectively. Reproduced from Ref. [73] under the Creative Commons Attribution 4.0 International License.

The hysteretic optical response of VO_2_ can also provide state retention in integrated photonic circuits. Figure 6 illustrates a VO_2_/Si memory in which optical programming and erasing pulses switch the device between two states on different branches of the transition hysteresis [73].

Materials integration is as important as optical design. VO_2_ growth and post-annealing require control of oxygen activity and temperature, which may be incompatible with completed complementary metal-oxide-semiconductor (CMOS) layers. Polycrystallinity, surface roughness, and interfacial reactions increase scattering and broaden the transition. Electrodes and heaters must be placed close enough for efficient actuation without adding optical absorption, and thermal isolation must reduce switching energy without making reset excessively slow. Encapsulation should preserve stoichiometry while maintaining a low-loss optical interface.

The most useful design objective is therefore not the largest standalone change in refractive index or resistance, but the best system-level compromise among extinction ratio, phase range, insertion loss, footprint, switching energy, speed, retention, endurance, and fabrication compatibility. Free-space metasurfaces benefit from large-area, spatially addressable control; guided-wave devices prioritise low loss and thermal isolation; absorbers and emitters require controlled angular and spectral response; and sensors require stability and calibration. Treating the optical mode, VO_2_ microstructure, activation mechanism, and heat-flow path as a coupled design problem is essential for translating a laboratory phase-transition contrast into a reliable nanophotonic device.

## 6. Performance Comparison

Performance comparisons among VO_2_ nanophotonic devices require care because the reported quantities are not always equivalent. A transmission switch, phase-only metasurface, perfect absorber, and thermal emitter do not share a single definition of modulation depth or insertion loss. Reported switching times may describe the initial electronic response, completion of the structural transition, the measured optical rise time, or the full write-reset cycle. Likewise, energy can denote incident optical-pulse energy, absorbed energy in VO_2_, electrical energy delivered to a heater, or energy consumed by the entire drive circuit. The comparisons below therefore retain the definitions used in the original experiments and mark unavailable quantities as not reported (NR), rather than converting them using unverified assumptions.

### 6.1. Operating Wavelength

The operating wavelength determines both the available material contrast and the suitable device architecture. In the visible portion, the transition modifies colour, scattering, and dielectric resonances, but absorption and the comparatively smaller useful index contrast constrain high-efficiency transmissive elements. Near-infrared devices, especially around 1.3–1.6 μm, benefit from mature silicon and silicon-nitride photonics and are attractive for compact waveguide switches and memories. The larger change in extinction coefficient at telecom wavelengths enables short absorption modulators, although it also creates substantial insertion loss [3,7].

In the short- and mid-wave infrared, the optical contrast is stronger and coincides with atmospheric, sensing, and thermal-imaging bands. Resonant absorbers have been demonstrated around 2.3–2.6 μm [66], while electrically controlled metasurfaces have reached transmission ratios of approximately 100 at 3.9 μm [54]. Across 8–14 μm, VO_2_ structures have enabled multilevel emissivity control and switchable polarisation imaging [70,72]. At terahertz frequencies, the phase-dependent conductivity dominates the response, enabling large changes in resonance damping and transmission [24,62]. Consequently, a performance table should state both the probe wavelength and, for optical activation, the pump wavelength.

### 6.2. Modulation Depth

For an intensity quantity, *X*, such as transmittance, reflectance, absorptance, or emissivity, a normalised modulation depth can be written as

(7) $$M_{X}=\frac{\left|X_{1}-X_{2}\right|}{\max(X_{1},X_{2})}\times 100\%,$$

but this definition should not replace the convention used in the cited experiment. Guided-wave devices are commonly compared using extinction ratio in decibels, as defined in Equation (5), whereas metasurfaces may report a transmission ratio, absolute phase shift, polarisation extinction ratio, or change in absorption. For example, a transmission ratio of 100 corresponds to 20 dB, but an absorptance change from 90% to 20% is an absolute change of 70 percentage points and a normalised depth of 77.8%; these descriptions are not interchangeable.

Table 5, Table 6, Table 7 and Table 8 separate free-space devices, guided-wave devices, adaptive emitters, and photonic memories. This architecture-based organisation avoids comparing an absorber’s emissivity with a waveguide’s insertion loss or a memory’s retention. Per-unit-length values are retained for waveguides because multiplying them by the active length is valid only when coupling and mode conversion remain unchanged. NR denotes a quantity that was not reported in the source, and N/A denotes a metric that is not applicable to that architecture.

### 6.3. Insertion Loss

Insertion loss is most meaningful for a nominally transmitting switch and must be quoted for the intended on state. In an absorption modulator, stronger overlap between the optical mode and VO_2_ increases extinction ratio per unit length but usually increases on-state loss as well. This trade-off is evident in the embedded waveguide, which obtains approximately 10 dB contrast within only 0.5 μm but has an insertion loss of approximately 6.5 dB [3,63]. Evanescently coupled devices can reduce local loss, but generally require a longer VO_2_ patch and therefore a larger heated volume.

For resonant devices, the reported loss also contains coupling loss, resonance detuning, scattering, and absorption in electrodes or metallic antennas. The appropriate system metric may instead be peak transmission, reflection efficiency, aperture efficiency, or diffraction efficiency. Absorbers and emitters should not be assigned an insertion loss; their useful metrics are absorption or emissivity contrast, linewidth, bandwidth, angular response, and parasitic emission outside the target band. Authors should state whether fibre-to-fibre coupling losses have been removed and whether IL refers to the complete device, the active section alone, or a value normalised by length.

### 6.4. Switching Time and Energy

The turn-on time $t_{\mathrm{on}}$ and recovery time $t_{\mathrm{off}}$ should be reported separately. Femtosecond pump-probe experiments and hybrid waveguides can resolve a sub-picosecond initial optical response [77], whereas thermally completed devices commonly recover on nanosecond-to-microsecond timescales. Optically monitored, electrically driven devices have shown turn-on below 2 ns [65], but this does not imply a multi-gigahertz steady-state modulation rate because cooling and structural recovery can be slower.

For an electrical drive, the energy delivered during a transition is

(8) $$E_{\mathrm{sw}}=\int_{t_{0}}^{t_{1}}V\left(t\right)I\left(t\right)\;dt,$$

whereas an optical experiment should distinguish incident pulse energy from the fraction absorbed in the active VO_2_. A useful thermal lower-bound estimate is

(9) $$E_{\mathrm{th}},\min≃V_{\mathrm{act}}\left[C_{V}\left(T_{\mathrm{MIT}}-T_{0}\right)+L_{V}\right],$$

where $V_{\mathrm{act}}$ is the transformed volume, $C_{V}$ is volumetric heat capacity, and $L_{V}$ is latent heat per unit volume. The actual system energy is larger because the substrate, cladding, electrodes, and heater are also heated.

The scale of the architecture is therefore decisive. An all-optical Si_3_N_4_/VO_2_ waveguide reported 6.4 pJ switching energy [74], while an in-plane-pumped Si/VO_2_ device required 15.8 nJ and switched in approximately 0.1–1 μs, with the fastest measured response near 318 ns [75]. A later 1-μm-long silicon photonic memory switched with a 24 pJ optical pulse, with measured write and erase times of approximately 24 and 36 ns, respectively [73]. These values cannot be compared without the active volume, pump geometry, absorbed fraction, holding power, and reset condition.

The architecture-separated comparisons in Table 6 and Table 8 retain the reported incident energy, write and reset times, holding power, and endurance protocol. Values not provided by the original report remain marked NR rather than being reconstructed from unverified thermal or optical assumptions.

### 6.5. Transition Temperature and Hysteresis

The transition temperature of bulk-like stoichiometric VO_2_ is near 341 K, or approximately 68 °C [4]. A nanophotonic device, however, does not have a universal transition temperature. Oxygen stoichiometry, dopants, epitaxial strain, crystallographic orientation, grain size, interfaces, and temperature gradients shift or broaden the measured response. Moreover, the transition extracted from resistance may not coincide exactly with that extracted from transmission at a particular wavelength.

For comparison, the heating and cooling transition temperatures should be reported separately. If $T_{↑}$ and $T_{↓}$ denote consistently defined midpoints, then

(10) $$T_{c}=\frac{T_{↑}+T_{↓}}{2},\;\Delta T_{\mathrm{hyst}}=T_{↑}-T_{↓}.$$

The extraction criterion must accompany these values, whether it is the midpoint, maximum derivative, onset, or completion temperature. In the in-plane-pumped waveguide, for example, the heating transition occurred over approximately 55–63 °C, whereas the cooling response extended over a much broader interval [75]. Hysteresis is undesirable for an analog modulator requiring a single-valued transfer function, but it can be deliberately used for bistability and memory [73,76]. Lowering $T_{\mathrm{MIT}}$ reduces the temperature excursion and energy required for thermal switching; however, it also brings the transition closer to the normal operating temperature. The device then becomes more susceptible to ambient-temperature fluctuations and self-heating, and the temperature range over which the insulating off state remains stable becomes smaller. The appropriate $T_{\mathrm{MIT}}$ should therefore be set relative to the highest specified ambient and self-heating temperature, rather than simply being placed as close as possible to room temperature.

### 6.6. Endurance and Device Architecture

Endurance is the number of complete write-erase cycles over which the specified optical contrast, threshold, and timing remain within tolerance. It should not be inferred from electrical cycling of an unpatterned film. Optical devices can fail through stoichiometric drift, oxidation, interdiffusion, cracking, delamination, heater or electrode damage, permanent filament formation, and progressive loss of array uniformity. The test waveform, duty cycle, ambient temperature, encapsulation, holding bias, and failure criterion must therefore be reported.

Architecture strongly affects endurance. Large-area films and resonant absorbers are easy to characterise spectrally but require heating a comparatively large thermal mass. Metal heaters offer electrical integration yet introduce optical loss and local hot spots. Evanescent waveguide patches reduce the transformed volume and permit rapid readout, while suspended devices reduce energy at the expense of cooling speed and mechanical robustness. Separate heaters avoid direct current crowding in VO_2_, whereas direct-contact devices are compact but more susceptible to filamentary switching.

The progress in integrated memories illustrates why endurance must be measured directly. A voltage-biased hybrid memory demonstrated stable operation beyond 4000 cycles [76]. A later VO_2_/Si memory preserved its device performance through as many as $10^{7}$ write-erase cycles; at a separate best-performance operating point it used a 24 pJ pulse and produced 24 ns write and 36 ns erase times [73]. During the endurance measurement, however, the device used 1 μs programming pulses and 180 μW holding power. Quoting all four values without this protocol distinction would incorrectly imply that the endurance record was obtained simultaneously at the minimum-energy, fastest operating point.

No single architecture is best across all metrics. Resonant metasurfaces maximise free-space modulation within a thin optical path but trade bandwidth and tolerance; nonresonant waveguides offer broadband operation but trade extinction against loss; thermal emitters prioritise emissivity, angular response, and stability rather than IL; and hysteretic memories exchange nonvolatility or holding power for high cycling speed. A credible performance comparison should therefore give the measured operating wavelength and polarisation, active dimensions, optical metric and its definition, on-state IL, separate write and reset times, incident and absorbed switching energy, holding power, $T_{↑}$ and $T_{↓}$, endurance protocol, and complete device architecture.

## 7. Challenges and Outlook

The large and reversible optical contrast of VO_2_ has enabled compelling device demonstrations, but material contrast alone does not determine whether a nanophotonic platform is practical. Optical loss, transition temperature, film variability, thermal overhead, process compatibility, and manufacturing yield are strongly coupled. For example, increasing the optical overlap with VO_2_ improves extinction but generally increases insertion loss, while thermal isolation reduces switching energy but slows reset and increases thermal cross-talk. The most credible path forward is therefore the co-design of the VO_2_ film, optical mode, actuator, and thermal environment rather than the optimisation of any one quantity in isolation. Table 9 summarises the principal trade-offs.

### 7.1. Optical Loss

VO_2_ is intrinsically absorptive over much of the visible and infrared spectrum, and its metallic phase has a substantial free-carrier contribution to the extinction coefficient [3,7]. This absorption is useful in compact amplitude modulators and switchable emitters, but it is detrimental to transmissive switches, phase shifters, and cascaded photonic circuits. The central device-level compromise is therefore the overlap between the optical mode and VO_2_: stronger overlap increases extinction ratio per unit length, yet also increases the loss in the nominally transmitting state. An embedded VO_2_/Si waveguide illustrates this limit, providing approximately 10 dB of contrast within a 0.5-μm active length but with approximately 6.5 dB insertion loss [3,63]. Polycrystalline roughness, grain boundaries, imperfect patterning, and absorbing electrodes add extrinsic loss to this material floor [64].

Loss mitigation should be architecture-specific. Evanescent coupling and thinner VO_2_ patches reduce absorption but require longer interaction lengths or stronger resonant enhancement. Hybrid dielectric resonators can concentrate the field near, rather than throughout, the VO_2_, and interferometric or phase-dominant designs can convert a refractive-index change into intensity modulation with less absorbing material. Resonance can nevertheless narrow the bandwidth and amplify sensitivity to thickness and temperature variations. Future reports should therefore provide total device throughput, on-state insertion loss, the extinction ratio, the active length or area, bandwidth, and the contributions of couplers, electrodes, and the VO_2_ section separately. A record extinction ratio is of limited system value if it is obtained at negligible transmitted power.

### 7.2. High Transition Temperature

Bulk-like stoichiometric VO_2_ switches near 341 K, or approximately 68 °C [4]. This temperature is accessible in laboratory devices, but maintaining a photonic circuit above ambient increases energy consumption, thermal drift, and stress on adjacent materials. Lowering the transition temperature can reduce the required temperature excursion; however, it also reduces the margin against unintentional switching under ambient or self-heating fluctuations. The optimum transition temperature is consequently application-dependent rather than universally equal to room temperature.

W doping is among the most effective demonstrated routes. Atomic-layer-deposited W-doped VO_2_ on 200-mm Si wafers reached a transition temperature of approximately 32 °C at 1.63 at.% W and retained an emissivity contrast above 40% in a thermal-emitter structure [34]. The same study showed the associated compromise: increasing W content made the transition more gradual and reduced the optical contrast. Mo and Nb doping, oxygen nonstoichiometry, and epitaxial strain provide further tuning degrees of freedom [35,36,43] but can alter carrier concentration, hysteresis, loss, and long-term stability. Transition engineering should therefore report the heating and cooling thresholds together with optical constants in both phases, rather than presenting the reduced threshold alone. For many devices, a transition moderately above the highest specified ambient temperature may offer a better balance of standby stability and switching energy than an aggressively depressed room-temperature transition.

### 7.3. Film Uniformity and Phase Purity

The synthesis window for phase-pure VO_2_(M1) is narrow because vanadium supports several stable oxidation states and competing phases, including V_2_O_3_, V_2_O_5_, and Magnéli or Wadsley oxides. Small variations in oxygen activity can therefore change the carrier density and suppress or broaden the transition [12,13]. Thickness, grain size, crystallographic orientation, strain relaxation, and interface chemistry add further spatial variability [14,44]. In a resonant nanophotonic array, these variations appear as pixel-to-pixel shifts in resonance, switching threshold, hysteresis, and optical contrast even when the average film is nominally phase-pure.

Process development should combine structural, chemical, optical, and functional measurements. Diffraction and Raman spectroscopy identify crystalline phases; X-ray photoelectron spectroscopy probes oxidation state; ellipsometry measures the complex optical response; and spatially resolved temperature sweeps reveal distributions of transition temperature and hysteresis. A wafer-scale combinatorial growth study demonstrated why such mapping is important: a 3-inch epitaxial VO_2_ film achieved 1.2% thickness variation, four orders of magnitude resistance change, and mapped transition-temperature standard deviations below 1 °C across the wafer [78]. Buffer or seed layers, controlled post-annealing, and encapsulation can improve nucleation and suppress environmental degradation [42]. Nevertheless, phase purity measured at one or two locations is insufficient evidence of device-scale yield; future studies should publish wafer maps and distributions across patterned devices.

### 7.4. Device Heating and Energy Consumption

In a thermally activated device, the energy cost is not limited to the latent and sensible heat of the transformed VO_2_. The substrate, cladding, electrodes, and heater also absorb energy, often dominating the thermal mass. Increasing thermal resistance reduces the power required to reach the transition, but it generally lengthens the cooling time and increases cross-talk between neighboring elements. Conversely, aggressive heat sinking accelerates reset while increasing write power. The thermal conductivity and heat capacity also vary through the transition, so a single constant-property thermal model can misestimate both threshold and dynamics [41].

The strongest near-term reductions in energy are likely to come from shrinking the switched volume and heating it selectively. Short waveguide patches, nanoscale heaters, pulsed drive, and suspended or undercut structures can reduce parasitic heating. Optical resonances can localise absorbed pump power, while separate heaters can avoid current crowding and filament formation inside VO_2_. Each approach introduces a corresponding cost: suspension can reduce mechanical robustness, resonant heating narrows spectral tolerance, and direct metallic heaters add optical absorption. Published device metrics already span from picojoule-scale pulses in micron-scale photonic memories to nanojoule operation in larger pumped waveguides [73,75]; these values must be interpreted together with active volume, absorbed fraction, holding power, reset time, and drive electronics. Ionic, electrochemical, strain, or ferroelectric control may reduce steady-state heating, but they introduce separate constraints on speed, retention, chemical reversibility, and endurance. Thus, the relevant target is minimum system energy per complete and repeatable write-reset event, not minimum trigger energy in isolation.

### 7.5. CMOS-Compatible Integration

The phrase “CMOS-compatible” requires a precise process definition. Depositing VO_2_ on Si or SiO_2_/Si demonstrates substrate compatibility, but does not by itself establish compatibility with front-end transistors, back-end metallisation, or a completed photonic wafer. VO_2_ growth and crystallisation can require elevated temperatures and controlled oxygen exposure; both may exceed the thermal or chemical budget of existing metals, low-*k* dielectrics, and contacts. Vanadium cross-contamination, etch selectivity, film roughness, contact resistance, and oxidation during later processing must also be considered.

Atomic layer deposition is attractive because it offers thickness control and conformal coating, and W-doped VO_2_ has been deposited at 200 °C on 200-mm Si wafers, followed by a 400 °C crystallisation anneal [34]. More recent pulsed laser deposition (PLD) and ALD work has produced continuous functional films as thin as 6–8 nm on thermally oxidised Si, but the optimised processes still used growth or annealing steps around 400–500 °C [28]. These results are important integration advances, yet they also show why deposition temperature alone is an incomplete compatibility metric. Promising routes include rapid or localised laser annealing, diffusion and oxidation barriers, wafer bonding or membrane transfer, and post-fabrication placement of a small VO_2_ volume on passive photonic circuits. Future work should identify the exact integration level, maximum time-temperature exposure, allowed ambient, contamination controls, lithographic sequence, and compatibility with foundry design rules.

### 7.6. Large-Area Fabrication

Scaling a film and scaling a nanophotonic device are distinct problems. A uniform blanket film must first provide controlled thickness, stoichiometry, roughness, optical constants, transition temperature, and hysteresis. Pattern transfer must then preserve critical dimensions and sidewall quality across the wafer, while heaters, contacts, and addressing lines must deliver reproducible local temperature. The 3-inch combinatorial-growth result demonstrates wafer-scale phase and transition uniformity on sapphire [78]; ALD has extended VO_2_ and W-doped VO_2_ deposition to 200-mm Si wafers with approximately 4% thickness nonuniformity [34]. Spray pyrolysis and epitaxial self-patterning offer potentially scalable material and metasurface routes [10,16], although uniform nanophotonic linewidth, actuator integration, and array yield remain to be established at manufacturing scale.

Large-area progress should therefore be assessed statistically. Useful reporting includes across-wafer maps of thickness and optical constants, distributions of heating and cooling thresholds, resonance variation after patterning, switching yield, dead-pixel fraction, and drift after cycling. Stepper photolithography, interference lithography, or nanoimprint processes may be more scalable than serial electron-beam patterning, but they require designs tolerant to linewidth and material variations. Redundant addressing, local calibration, and closed-loop temperature control may compensate for residual nonuniformity in adaptive emitters or imaging arrays, although such circuitry adds area and power.

The near-term priority for VO_2_ nanophotonics is thus reproducibility at the device and wafer levels rather than another isolated record value. Standardised temperature-dependent optical-constant datasets, explicit switching-energy boundaries, separate write and reset dynamics, environmental and cycling tests, and distribution-based reporting would make results more comparable. In parallel, electromagnetic, thermal, mechanical, and materials optimisation should be performed jointly. VO_2_ is most likely to succeed where its unusually large, broadband, and reversible optical change justifies some absorption and thermal overhead, for example, compact free-space modulators, adaptive infrared emitters, nonvolatile or hysteretic photonic elements, and functions that are difficult to obtain from conventional electro-optic materials. Demonstrating low-loss operation, controlled local activation, CMOS-relevant process integration, and high-yield wafer-scale fabrication in the same platform remains the decisive next step.

## Figures and Tables

**Table 1:** Comparison of the present review with selected previous VO_2_ reviews.

Review	Principal Emphasis	Relationship to the Present Work
Shao et al. (2018) [17]	Transition mechanism and modulation by doping, field, strain, and light	Provides the mechanism-level foundation; the present work traces defect location and interface boundary conditions into nanophotonic performance and reliability.
Liu et al. (2018) [18]	Phase physics, dynamics, phase diagrams, imperfection effects, and applications	Offers a broad physics-and-applications survey; the present review applies a defect/interface hierarchy and architecture-specific optical benchmarks.
Cueff et al. (2020) [3]	VO_2_ nanophotonic concepts, material integration, and device opportunities	Closest in photonic scope; the present review adds a defect- and interface-centred causal framework and explicit reporting of energy, speed, loss, and endurance conditions.
Zhang et al. (2021) [19]	Polymorphs, fabrication of films and low-dimensional structures, properties, and applications	Supplies broad fabrication and nanostructure context; the present review emphasises how local chemistry and boundaries govern usable optical contrast and cycling stability.
Bhupathi et al. (2023) [20]	Multi-stimulus response and application landscape	Complements the activation survey; the present work distinguishes electrostatic, ionic, thermal, optical, electrical, strain, and ferroelectric pathways by volatility, kinetics, reversibility, and endurance.
Wang et al. (2025) [21]	Switching dynamics of VO_2_-enabled optoelectronic devices	Provides a dynamics-centred update; the present review additionally links switching behaviour to defect/interface engineering and separates device architectures before quantitative comparison.
Present review	Defect and interface engineering for reconfigurable nanophotonics	Connects defect location, valence/stoichiometry, strain, contact and dielectric chemistry, oxygen exchange, and thermal boundaries to architecture-specific loss, threshold, energy, time, retention, and endurance.

**Table 2:** Complementary probes for defects and local phase state in VO_2_.

Method	Primary Information	Typical Sensitivity	Important Limitation
XPS/HAXPES	$V^{3+}$/V^4+^/$V^{5+}$ components, O 1s chemistry, depth trends	Surface sensitive for XPS; more bulk sensitive for HAXPES	Peak components are model dependent; sputtering and beam exposure can reduce VO_2_; valence fractions are not unique vacancy concentrations
EPR	Paramagnetic V centres and defect-associated trapped electrons	Bulk sensitive when a signal is detectable	Diamagnetic or EPR-silent charge states are missed; overlapping signals complicate absolute quantification
Raman spectroscopy	M1/M2/T/R fingerprints, V-V and V-O vibrations, strain, and disorder	Optical penetration depth and spot-size dependent	Vacancies are inferred indirectly; laser heating and mixed phases can shift or broaden peaks
XAS/XANES	Average valence, V-O hybridisation, orbital occupancy, and V-V dimer signatures	Surface-sensitive electron yield or more bulk-sensitive fluorescence	Spatial averaging and self-absorption; oxidation-state changes are not unique to oxygen vacancies
TEM/STEM-EELS	Interfaces, dislocations, grain boundaries, phase fronts, and nanoscale valence/oxygen maps	Atomic-to-nanometre scale	Limited sampling statistics; specimen preparation and the electron beam can alter oxygen content or phase

**Table 3:** Representative effects and trade-offs of VO_2_ dopants. Reported trends depend on concentration, deposition method, oxygen activity, strain, and microstructure.

Dopant	Nominal Valence/Site	Dominant Effect	Representative Transition Trend	Optical/Electrical and Stability Trade-Off
W	W^6+^ on V site	Strong electron donation and local lattice expansion	Strong decrease; approximately 32 °C at 1.63 at.% W in Ref. [34]	Broader transition and reduced phase contrast at higher content; possible clustering or secondary phases
Mo	Mo^6+^ on V site	Electron donation with local distortion different from W	Approximately linear decrease over 0–2% in Ref. [35]	Smoother hysteresis and stronger carrier backscattering; optical constants remain concentration dependent
Nb	Nb^5+^ on V site	Moderate donor; lattice and grain-structure modification	Generally decreases $T_{\mathrm{MIT}}$ [36]	Optimised content may enhance thermal sensing; excessive content can reduce resistance and optical contrast
Cr/Al/Ga/Fe	Predominantly trivalent substitution	Charge compensation, strain, and M2/T stabilisation	Often raises $T_{\mathrm{MIT}}$ or introduces multistep transitions	Greater phase complexity and hysteresis; potentially useful when higher-temperature stability is required
Ti or other near-isovalent species	Approximately tetravalent	Primarily elastic, structural, and microstructural effects	Direction depends on site occupancy, strain, and oxygen activity	May preserve carrier density better than donor doping but can disrupt V-V chains
H	Interstitial proton/electron donor	Chemical expansion and orbital filling; multiple H_*x*_VO_2_ phases	Can suppress the ordinary MIT or create an insulator-metal-insulator sequence	Nonvolatile or multilevel potential, but diffusion-limited speed and sensitivity to dose, catalyst, and atmosphere

**Table 4:** Comparison of electrostatic, electrochemical, and ionic-control mechanisms in VO_2_. Representative times are order-of-magnitude ranges only. Switching time and endurance are strongly device- and condition-dependent because they vary with film thickness, mobile species, electrolyte or gate dielectric, bias waveform, temperature, geometry, state criterion, and the definition of a completed write–reset cycle. NR denotes not reported or not standardised.

Mechanism	Material Change	Volatility/Retention	Representative Response Time	Reversibility/Endurance Evidence
Electrostatic accumulation	Interfacial carrier density only; no intended stoichiometry change	Volatile; state vanishes with bias	Potentially sub-μs, but no transferable range is established; RC limited	Standardised cycle count NR; limited by screening, leakage, charge trapping, and dielectric breakdown
O-vacancy formation or migration	${\mathrm{VO}}_{2-x}$ and $V^{3+}$-rich regions; lattice relaxation	Usually nonvolatile; long retention	Seconds to minutes in many gating experiments; strongly temperature dependent	Standardised cycle count NR; requires controlled reoxidation or back-migration and may suffer drift, roughening, or electrolyte reactions
Proton/H insertion	H_*x*_VO_2_ with electron donation and chemical expansion	Nonvolatile or quasi-nonvolatile	Commonly seconds to minutes in electrolyte or solid-proton-conductor demonstrations; thickness and dose dependent	Standardised cycle count NR; reversible only over a finite dose window, and high H content may stabilise a second insulating phase
Li^+^/Na^+^ or other ion intercalation	Ion-containing VO_2_ with changed orbital occupancy	Generally nonvolatile	Commonly seconds to minutes across unlike cells; diffusion and geometry dependent	Standardised cycle count NR; volume change, trapping, side reactions, and incomplete deintercalation can accumulate
Electrochemical reduction (general)	Redox-driven composition and valence change	Generally nonvolatile	Subsecond to minutes across unlike devices; not directly comparable	Standardised cycle count NR; the mobile species and recovery of the original chemistry must be demonstrated

**Table 5:** Representative free-space metasurfaces and absorbers. Incident and absorbed quantities are kept distinct; NR denotes not reported and N/A denotes not applicable.

Device	VO_2_ Thickness/Active Area	Probe; Activation/Temperature	Optical Metric	Throughput/Loss	Incident/Absorbed Energy or Power	$t_{\mathrm{on}}/t_{\mathrm{off}}$; Endurance
Au–VO_2_ resonant absorbers [66]	Hybrid nanowire/nanodisc arrays; VO_2_ thickness and active area NR	2.6/2.3 μm; stage heating above ∼68 °C	Absorptance 90% to 20% and 96% to 32%	N/A; reflective absorber	Incident/absorbed energy and holding power NR	$t_{\mathrm{on}}$/$t_{\mathrm{off}}$ NR; cycles NR
VO_2_–metal metasurface [54]	120-nm VO_2_; 400 μm × 400 μm array	3.9 μm; Au-heater current 150–310 mA; ambient start	Transmission ratio ∼100 (∼20 dB)	Open-state T∼0.5 design target; closed-state T∼0.005	Limiter threshold 20–180 mW incident; absorbed power NR	Simulated photothermal response ∼1–1000 μs; recovery/cycles NR
Polarisation-imaging metasurface [72]	VO_2_ metasurface; thickness/active area NR	8–14 μm; thermal; exact local temperature NR	Polarisation extinction ratio up to 10 dB	Maximum insulating-state transmission up to 65%	Incident/absorbed energy and holding power NR	$t_{\mathrm{on}}$/$t_{\mathrm{off}}$ NR; cycles NR

**Table 6:** Representative guided-wave switches and modulators. Energies are incident values unless explicitly identified as absorbed; NR denotes not reported.

Device	Active Dimensions	Signal/Pump	Modulation	Insertion Loss	Energy/Power	$t_{\mathrm{on}}/t_{\mathrm{off}}$ and Endurance
Embedded VO_2_/Si switch [3,63]	0.5 μm interaction length; thickness/volume NR	Signal ∼1550 nm; thermal activation; local *T* NR	9.7±0.8 dB broadband contrast	6.5±0.9 dB	Incident/absorbed energy and power NR	$t_{\mathrm{on}}$/$t_{\mathrm{off}}$ NR; cycles NR
Si_3_N_4_/VO_2_ all-optical modulator [74]	4 μm active length; VO_2_ thickness/volume NR	Signal 1500–1600 nm; pump 700–1000 nm; local *T* NR	1.68 dB μm^−1^	0.98 dB μm^−1^	6.4 pJ incident pulse; absorbed energy NR	$t_{\mathrm{on}}$/$t_{\mathrm{off}}$ NR; cycles NR
In-plane-pumped VO_2_/Si switch [75]	40-nm VO_2_; 20 μm patch; ∼15μm switched length	Signal 1550 nm; pump 1560 nm; heating branch ∼55–63 °C	14 dB measured (0.7 dB μm^−1^)	0.91 dB μm^−1^, insulating state	15.8 nJ incident pulse; 13.2 mW pump; absorbed energy NR	0.1–1 μs; fastest ∼318 ns; cycles NR

**Table 7:** Representative adaptive thermal emitters. Insertion loss is not applicable to an emitter; NR denotes not reported.

Device	VO_2_/Architecture	Spectral Band	Emissivity Metric	Activation/Temperature	Power/Dynamics/Endurance
Natural VO_2_ emitter [67]	∼150-nm epitaxial VO_2_ on sapphire	Mid-infrared; narrow plus broadband response	Near-blackbody feature and negative differential thermal emittance	Stage heating through the transition; >10 °C negative-differential-emittance interval	Heating power, $t_{\mathrm{on}}$/$t_{\mathrm{off}}$, and cycles NR
Transparent metasurface emitter [69]	40-nm VO_2_ metasurface with AZO reflector	Thermal infrared with visible transparency	State-dependent simulated and measured emissivity	Thermal; $T>T_{c}$ for hot state	NR
Multilevel emitter [70]	Spatially addressable VO_2_ emitter; thickness/active area NR	8–14 μm	Emissivity 0.19–0.91; up to nine levels	Thermal/electrical addressing; local temperature NR	Holding power, $t_{\mathrm{on}}$/$t_{\mathrm{off}}$, and cycles NR

**Table 8:** Representative integrated photonic memories. Best speed/energy and endurance values may have been measured under different protocols in the same study; NR denotes not reported.

Device	Active Dimensions	Programming/Readout	Energy/Holding Power	Write/Reset Time	Retention/Volatility	Endurance Protocol
Voltage-biased VO_2_/Si memory [76]	40-nm VO_2_ on a 220 nm × 500 nm Si waveguide; ∼5μm active length	Optical write with electrical bias; optical readout; local temperature NR	23.5 pJ minimum incident write pulse; absorbed energy/holding power NR	∼100 ns 10–90% rise; reset time NR	Hysteretic, bias-assisted state	>4000 cycles under the reported waveform
High-endurance VO_2_/Si memory [73]	40-nm VO_2_ on 220 nm × 500 nm Si; 1 μm speed device/3 μm endurance device	1550-nm write/erase and 1565-nm read; holding within thermal hysteresis	24 pJ incident at best-performance point; 180 μW holding in endurance test; absorbed energy NR	Write 12–28 ns; erase 36–208 ns; 24/36 ns at 24 pJ	Volatile hysteretic memory requiring a holding condition	Up to $10^{7}$ cycles; 1-μs write/erase pulses, 100-μs period, 180-μW holding

**Table 9:** Principal challenges for VO_2_ nanophotonics, promising mitigation routes, and the trade-offs that remain.

Challenge	Primary Origin	Promising Route	Residual Trade-Off or Required Metric
Optical loss	Intrinsic absorption, defects, grain boundaries, metals, and scattering	Evanescent coupling, reduced VO_2_ volume, dielectric resonators, and phase-dominant operation	Extinction-loss balance, device length, bandwidth, and total throughput
High transition temperature	Bulk transition near 341 K	Doping, strain, defect control, and local rather than global heating	Optical contrast, hysteresis, ambient margin, and stability
Film variability and phase purity	Narrow oxygen-process window and competing vanadium oxides	Controlled growth/annealing, buffer layers, encapsulation, and wafer-scale metrology	Spatial distributions of optical constants, transition threshold, and hysteresis
Heating and energy	Active film plus substrate, cladding, electrodes, and heater are heated	Small active volume, thermal isolation, pulsed drive, and efficient local heaters	Write/reset asymmetry, holding power, cross-talk, and endurance
CMOS integration	Thermal budget, oxygen exposure, contamination, patterning, and contacts	ALD or sputtering, barrier layers, localised annealing, and heterogeneous transfer	Compatibility with a specified process level, not only with a silicon substrate
Large-area fabrication	Coupled thickness, stoichiometry, pattern, and actuator nonuniformity	Wafer-scale deposition, stepper or imprint patterning, and statistical process control	Device yield and distributions, not only average film properties

## Data Availability

No new data were created or analysed in this study. Data sharing is not applicable to this article.
